# Dynamics of TCR repertoire and T cell function in COVID-19 convalescent individuals

**DOI:** 10.1038/s41421-021-00321-x

**Published:** 2021-09-28

**Authors:** Lingjie Luo, Wenhua Liang, Jianfeng Pang, Gang Xu, Yingying Chen, Xinrong Guo, Xin Wang, Yi Zhao, Yangdian Lai, Yang Liu, Bin Li, Bing Su, Shuye Zhang, Michal Baniyash, Lei Shen, Lei Chen, Yun Ling, Ying Wang, Qiming Liang, Hongzhou Lu, Zheng Zhang, Feng Wang

**Affiliations:** 1 Shanghai Institute of Immunology, Department of Immunology and Microbiology, State Key Laboratory of Oncogenes and Related Genes, Shanghai Jiao Tong University School of Medicine, Shanghai, China; 2 Research Center of Translational Medicine, Shanghai Children’s Hospital, Shanghai Jiao Tong University School of Medicine, Shanghai, China; 3 The Center for Microbiota and Immunological Diseases, Shanghai Institute of Immunology, Shanghai General Hospital, Shanghai Jiao Tong University School of Medicine, Shanghai, China; 4 Institute for Hepatology, National Clinical Research Center for Infectious Disease, Shenzhen Third People’s Hospital; The Second Affiliated Hospital, School of Medicine, Southern University of Science and Technology, Shenzhen, Guangdong China; 5 Shanghai Public Health Clinical Center, Fudan University, Shanghai, China; 6 The Lautenberg Center for Immunology and Cancer Research, Israel-Canada Medical Research Institute, Faculty of Medicine, The Hebrew University, Jerusalem, Israel

**Keywords:** Cell biology, Immunology

## Abstract

SARS-CoV-2 outbreak has been declared by World Health Organization as a worldwide pandemic. However, there are many unknowns about the antigen-specific T-cell-mediated immune responses to SARS-CoV-2 infection. Here, we present both single-cell TCR-seq and RNA-seq to analyze the dynamics of TCR repertoire and immune metabolic functions of blood T cells collected from recently discharged COVID-19 patients. We found that while the diversity of TCR repertoire was increased in discharged patients, it returned to basal level ~1 week after becoming virus-free. The dynamics of T cell repertoire correlated with a profound shift of gene signatures from antiviral response to metabolism adaptation. We also demonstrated that the top expanded T cell clones (~10% of total T cells) display the key anti-viral features in CD8^+^ T cells, confirming a critical role of antigen-specific T cells in fighting against SARS-CoV-2. Our work provides a basis for further analysis of adaptive immunity in COVID-19 patients, and also has implications in developing a T-cell-based vaccine for SARS-CoV-2.

## Introduction

The year 2019 ended with the emergence of severe acute respiratory syndrome coronavirus 2 (SARS-CoV-2)^1^. This disease was officially named coronavirus disease 2019 (COVID-19) by the World Health Organization^2^. Shortly after, COVID-19 outbreak spread globally and became a pandemic disease^3,4^. The genome sequence of SARS-CoV-2 bears 79.5% identity to that of SARS-CoV^5^. Like SARS-CoV and MERS-CoV, SARS-CoV-2 belongs to beta genus Coronavirus in Corornaviridae family^6^.

The T-cell-mediated immune response is one of the primary defense mechanisms of adaptive immune system against virus^7^. T cells orchestrate adaptive immunity following the signalling dictated by their clonotypic T cell receptors (TCRs), which recognize a peptide (8–15 amino acids) presented by major histocompatibility complex (MHC)^8^. Recognition of peptide-MHC complex (pMHC) by TCR induces activation and differentiation of naive T cells to various functional subsets during acute stages of infection and leads to the eradication of invading pathogens^9^.

Both chains of TCR (α and β) consist of a variable (V) region, junctional (J) region, and constant (C) region. The diversity (D) region connects V and J regions and forms an integral β chain^10^. Thus, TCR recombination process generates highly diverse complementarity‐determining regions (CDRs) localized in the TCR α and β chains, forming a functional and highly diverse TCR repertoire. The CDR sequences determine the specificity of TCR binding to pMHCs, of which the third CDR (CDR3) is the most hypervariable and contributes to direct peptide recognition. During viral infection, CD8^+^ T cells recognize viral peptides and mediate killing of infected cells by releasing granzymes and perforin^9^.

Although considerable efforts have been spent on clarifying the immune response during SARS-CoV-2 infection^11,12^, little is known about the responses of antigen-specific T cells with their diverse TCR repertoire in human for targeting the virus. The immune system generates SARS-CoV-2-reactive T cells against the virus at the beginning of an acute respiratory distress syndrome in COVID-19 patients, and T cells are increased with time^13^. However, initial data from the clinical examinations of severe COVID-19 patients showed that both CD4^+^ and CD8^+^ T cells are decreased along with the deterioration of patients’ status^14^. Evidence suggests that the SARS-CoV-2 specific T cells are found in both COVID-19 patients and unexposed healthy donors^15,16^. In addition, it was shown that T cells respond to nucleocapsid protein (NP) of SARS-CoV-2 in both the SARS-recovered patients and uninfected individuals, suggesting that the memory response could be induced by previous infections of “common cold” human coronaviruses^16,17^. Moreover, the T cells from SARS-CoV-2 patients recognize multiple domains of NP, and the long-lasting memory T cells from 2003 SARS patients could respond to SARS-CoV-2 NP^18^. Thus, the anti-SARS-CoV-2 T-cell-mediated immune response could be crucial for the immune memory against SARS-CoV-2 infection. Nowadays, data are lacking regarding the dynamics of the generated TCR clones and the associated functional changes in these T cells, which are major players in the anti-viral immune processes. Here, we collected blood samples from COVID-19 patients who have recently become virus-free at different time points (Discharged *vs*. Follow-up). We performed a comprehensive single-cell analysis to examine both the TCR sequence and functional gene expression in these COVID-19 convalescent subjects.

## Results

### Global profile of CDR3 length and TCR V/J distribution

**Fig. 1:**
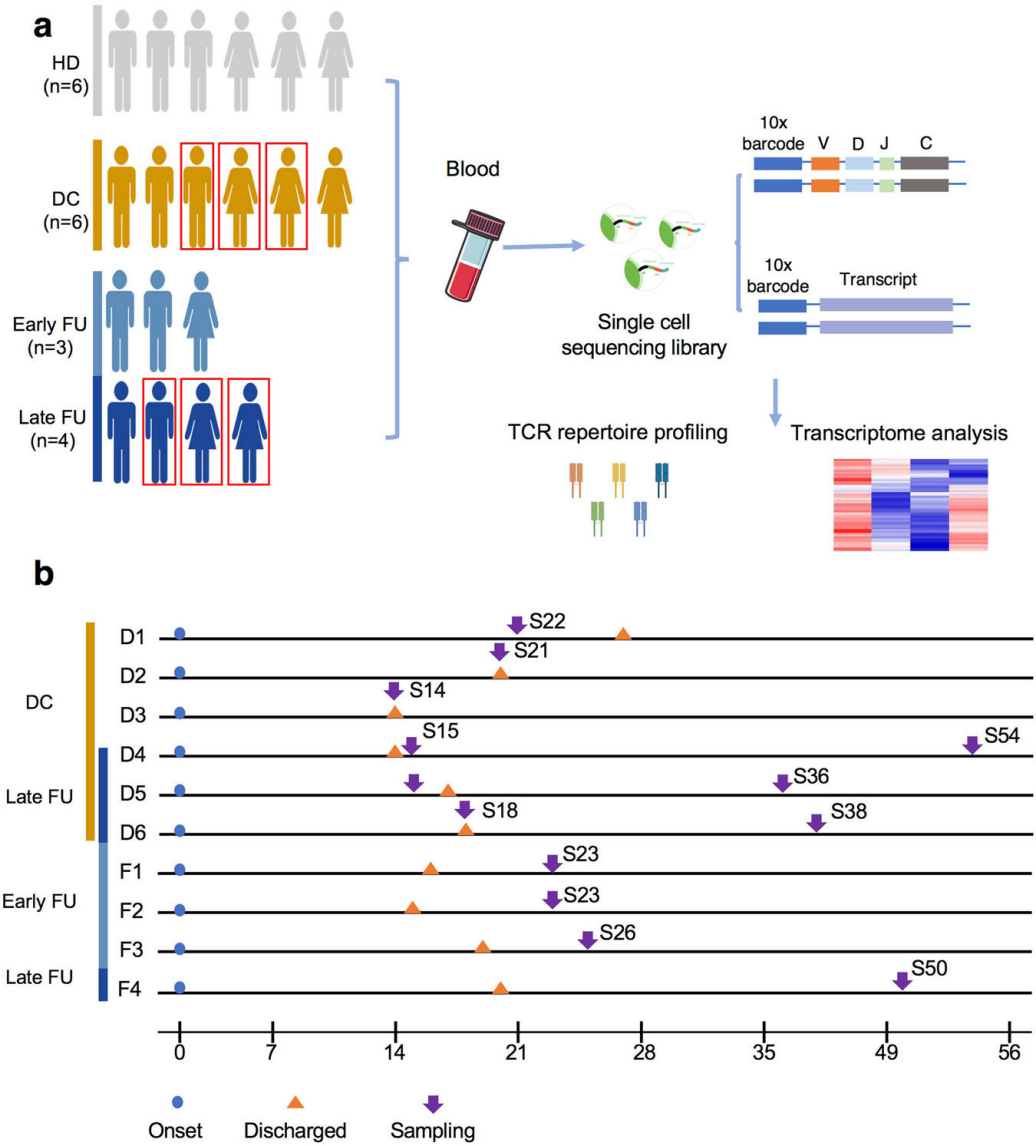
Study workflow. **a** A schematic workflow of study design. PBMCs were collected and separated from four groups: Discharged (DC), Early Follow-up (Early FU), Late Follow-up (Late FU), and Healthy Donor (HD). The Early FU and Late FU were divided from Follow-up group. PBMCs were applied for scRNA and scTCR sequencing using 10X-Based Genomics platform. The red box indicated the same patients at different stages in our study. **b** Timeline of the disease course for 10 patients infected with SARS-CoV-2. The color bars on the left represent groups with the same color as in (**a**). DC, Discharged group; FU, Follow-up group; HD, Healthy Donor group.

To reveal the TCR repertoire dynamic during immune responses to SARS-CoV-2, we assessed 10 recovered patients, in which all the patients were positive to SARS-CoV-2 nucleic acid test during hospitalization and had negative results in a SARS-CoV-2 nucleic acid test on discharge day. For the Discharged group, six of them were sampled 6 days before and 1 day after the discharge day. We further divided the Follow-up group into two subgroups. In particular, three patients were sampled on 7 days post-discharge (Early Follow-up), and four patients were samples between 19 and 40 days post-discharge (Late Follow-up), in which three patients were re-sampled from the Discharged group (Fig. 1, Supplementary Table S1). Moreover, peripheral blood mononuclear cells (PBMCs) from six healthy donors with the negative nucleic acid test were collected for control (HD). We isolated PBMCs to construct cDNA libraries and performed TCR-α/-β transcriptional sequencing analysis using 10X Genomic Single-cell-based platform (Fig. 1).

**Fig. 2:**
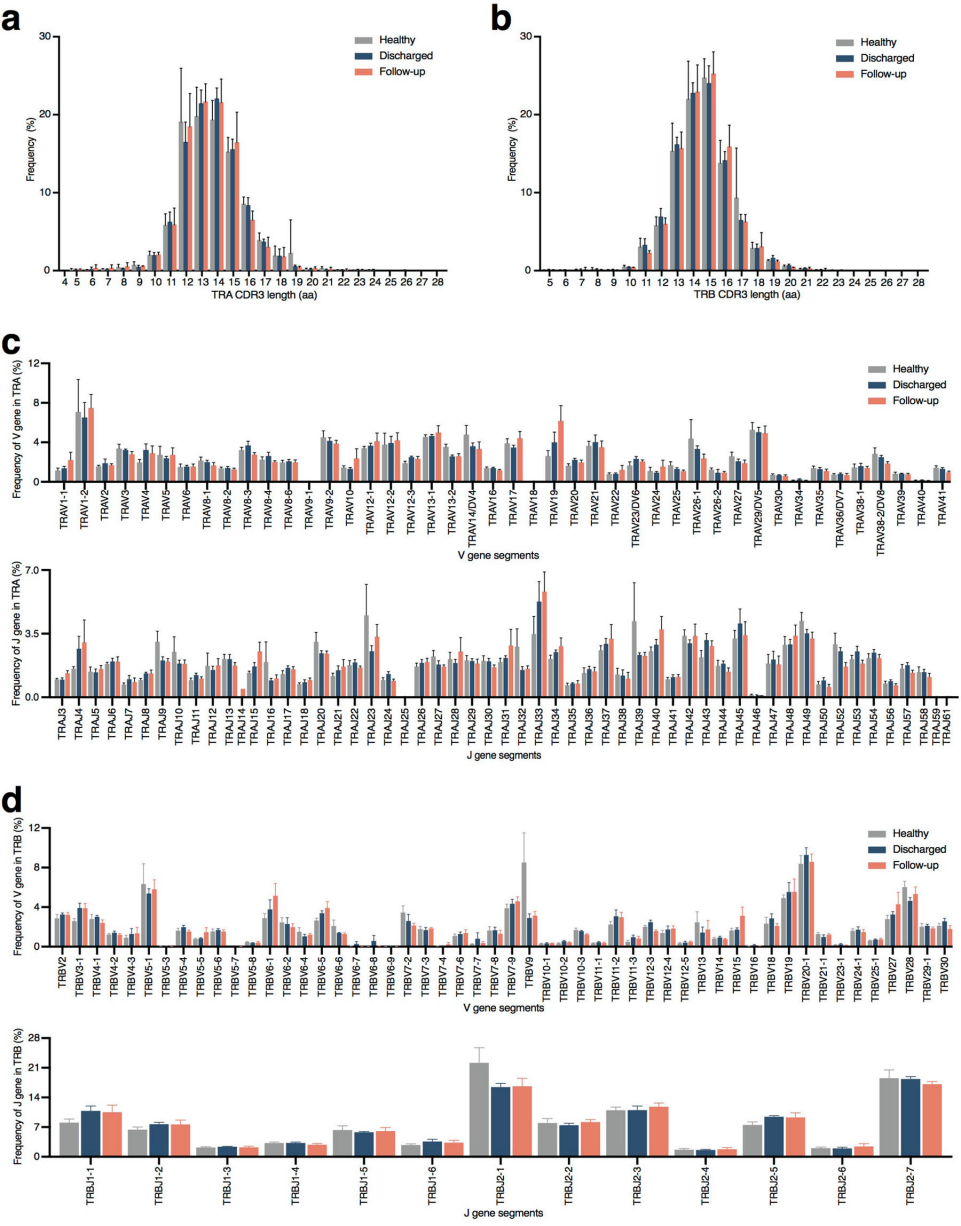
Global CDR3 length and V/J distribution in samples. The amino acid length of CDR3 from TCR α chain (**a**) and TCR β chain (**b**) of HD group, Discharged group and Follow-up group (*n* = 6 in Discharged and HD groups, *n* = 7 in Follow-up group). The usage rate of V and J regions from TCR α chain (**c**) and TCR β chain (**d**) of HD group, Discharged group and Follow-up group (*n* = 6 in Discharged and HD groups, *n* = 7 in Follow-up group). HD, Healthy Donor group.

We first assessed CDR3 length distribution across the TCR α and β CDR3 sequences from the HD, Discharged, and Follow-up groups to map the T cell repertoire diversity. The CDR3 length of the HD group floated between 5 and 28 aa with a peak of 19.76% cells at 13 aa of TCR α chain. The Discharged group displayed TCR α chain CDR3 length between 4 and 27 aa and a peak of 22.03% cells at 14 aa of TCR α chain. In the Follow-up group, the TCR α chain possessed 5 to 28 aa with the peak at 13 aa (21.62% cells) (Fig. 2a). The TCR β chain CDR3 length was between 5 and 26 aa in the HD group, 5 and 27 aa in the Follow-up group, and 5 and 28 aa in the Discharged groups. The TCR β chain CDR3 length of the three groups was similar with 24.71% cells from the HD group, 23.99% cells from the Discharged group, and 25.21% cells from the Follow-up group at the peak of 15 aa (Fig. 2b).

We then evaluated the usage of TCR V/J repertoires in healthy donors and recovered patients. Our data showed a high level of TRAV1-2 and TRAJ23 expression in the HD group. Both recovered groups exhibited increased expression of TRAV1-2 and TRAJ33 in the TCR α chain construction (Fig. 2c). Remarkably, TRBV20-1 was the top used TRBV gene in recovered patients, with the same high expression pattern of TRBJ2-7 (Fig. 2d, Table 1). While the top used TRBV and TRBJ were TRBV9/TRBV20-1 and TRBJ2-1/TRBJ2-7 in the HD group. Taken together, our result showed a similar CDR3 length and TCRV/J usage frequency in the groups of healthy donors and recovered patients.

**Table 1:** The highest TRAV/J usage in each group.

Pt#	TRAV	TRAJ	TRBV	TRBJ
HD	TRAV1-2	TRAJ23	TRBV9	TRBJ2-1
Discharged	TRAV1-2	TRAJ33	TRBV20-1	TRBJ2-7
Follow-up	TRAV1-2	TRAJ33	TRBV20-1	TRBJ2-7

### Discharged group displayed a higher diversity of TCR repertoire and a lower level of TCR clonal expansion

**Fig. 3:**
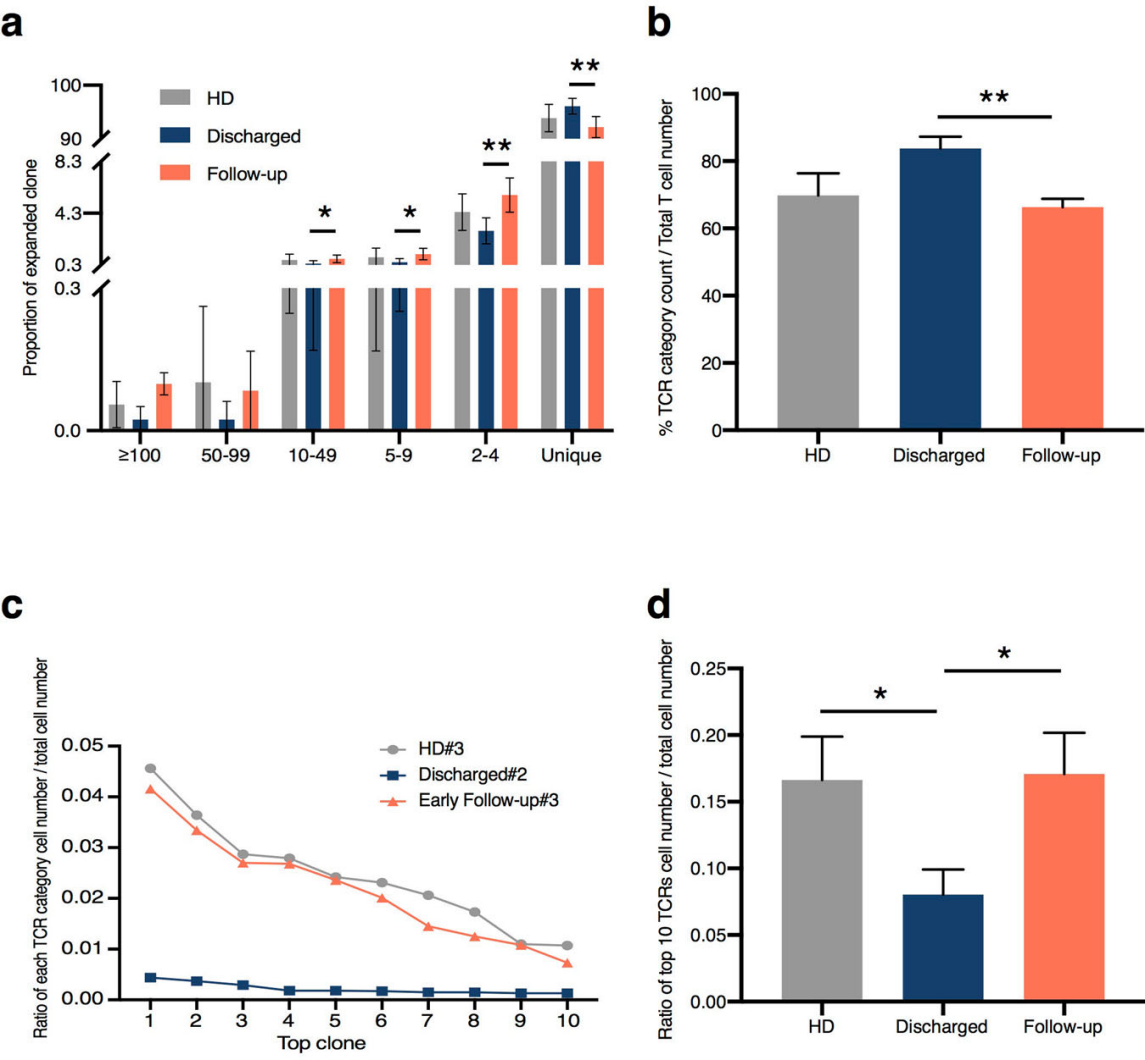
Clonal diversity and expansion. **a** The proportions of expanded clone ≥100 times expansion, 50 to 99 times expansion, 10 to 49 times expansion, 5 to 9 times expansion, 2 to 4 times expansion and not expanded (unique) in each group (*n* = 6 in Discharged and HD groups, *n* = 7 in Follow-up group). **b** The clone diversity of three groups, which was increased in the Discharged group (*n* = 6 in Discharged and HD groups, *n* = 7 in Follow-up group). **P < 0.01 by unpaired student’s test. **c** The ratio of cell number in each TCR classification to total cell number in top 10 TCR from representative samples. Ratio = cell count of each TCR classification/total cell count. **d** The ratio of top 10 TCRs’ cell number to total cell number from all samples, ratio = cell count of top 10 TCRs/total cell count (*n* = 6 in Discharged and HD groups, *n* = 7 in Follow-up group). *P < 0.05 by unpaired student’s test. HD, Healthy Donor group.

In order to further understand the dynamics of T cell repertoire post-SARS-CoV-2 infection, we analyzed the feature of T cell clonal expansion in the three groups. The percentage of TCR classification count (the number of different TCR sequences in a sample) to total cell number was used to measure the TCR diversity. This percentage in the representative sample of the Discharged group (#2) was much higher (94.51%) than the other two examples (53.75% in HD#3 and 61.21% in Follow-up#3) (Supplementary Fig. S1a). Moreover, the decreased proportion of high expanded clone (≥100), moderately expanded clone (10–49), small expanded clone (5–9), rarely expanded clone (2–4), and increased non-expanded clone (unique) fraction revealed higher clonotype diversity in the Discharged group (Fig. 3a, Supplementary Fig. S1b). In addition, we found a majority (82.95%) of non-expanded unique T cells from the Discharged group compared to the HD and Follow-up groups.

We next calculated the TCR diversity in all the three groups. We found that the Discharged group showed a higher level of TCR diversity at 83.80%, whereas this percentage was similar in both the HD group at 69.83% and the Follow-up group at 66.33% (Fig. 3b). Consistently, we found a significantly higher population of expanded T cells in the HD (32.87%) and the Follow-up (35.98%) groups compared with 17.05% in the Discharged group (Supplementary Table S5). We then calculated the cell number ratio in each TCR clonotype to total cell number from the representative healthy donor and recovered patient groups. The proportion of the top 10 TCR clonotypes in HD#3 and Follow-up#3 exhibited comparable tendency, whereas the Discharged#2 kept a persistently lower ratio with its top 10 TCR clonotypes (Fig. 3c). Consistently, a lower proportion of top 10 TCRs in total TCR repertoire was found in the Discharged group (0.0803), compared with 0.1709 in the Follow-up group (Fig. 3d). These data demonstrated that patients in the Discharged group showed a higher diversity and a lower level of clonal expansion of T cells in their blood post-SARS-CoV-2 infection.

### V/CDR3/D/J segments usage in the top 10 TCR clonotypes and global V–J pairing distribution analysis

The representative samples showed similar high expression of TRAV12, TRAJ23, TRAJ33, TRBV20-1, TRBJ2-1, and TRBJ27 in the top 10 TCR clonotypes highest used V/J genes in the three groups (Tables 2–4). A dramatic decrease in the frequency of top 10 clones was observed in the Discharged group compared to the HD and Follow-up groups. We next used a global V–J pairing distribution analysis to display the TCR features from three groups (Supplementary Fig. S2). Several dominant connections were observed in HD and Follow-up groups, including the TRAV1-2, TRAJ16, TRBV20-1, and TRBJ2-1 in HD group, TRAV1-2 TRAJ33, TRBV20-1, and TRBJ27 in the Follow-up group (Supplementary Fig. S2a, c). The Discharged group had a relatively uniform connection between different V–J combinations, consistent with an increased level of TCR diversity in this group (Supplementary Fig. S2b).

**Table 2:** Top 10 TCR clonotypes information of representative sample from HD group (HD#3).

TRAV	TRA-CDR3	TRAJ	TRAC	TRBV	TRB-CDR3	TRBJ	TRBC	Ratio
TRAV1-2	CAVRDEDGQKLLF	TRAJ16	TRAC	TRBV13	CASSLWGANEQFF	TRBJ2-1	TRBC2	4.56%
TRAV9-2	CAQRNTGGFKTIF	TRAJ9	TRAC	TRBV7-2	CASSSEQNQPQHF	TRBJ1-5	TRBC1	3.64%
TRAV5	CAESITGKLVF	TRAJ47	TRAC	TRBV20-1	CSASSGGEQFF	TRBJ2-1	TRBC2	2.83%
TRAV5	CAEISDGQKLLF	TRAJ16	TRAC	TRBV20-1	CSASQPGLVSREQFF	TRBJ2-1	TRBC2	2.79%
TRAV26-1	CIVRSTGGGADGLTF	TRAJ45	TRAC	TRBV20-1	CSASGGLVLDTQYF	TRBJ2-3	TRBC2	2.42%
TRAV14/DV4	CAMRDKQSNFGNEKLTF	TRAJ48	TRAC	TRBV19	CASMPRVAKNIQYF	TRBJ2-4	TRBC2	2.31%
TRAV13-2	CAENFSNTGTASKLTF	TRAJ44	TRAC	TRBV5-1	CASSPARTGPYNEQFF	TRBJ2-1	TRBC2	2.06%
TRAV21	CAVNDQGGKLIF	TRAJ23	TRAC	TRBV10-3	CAISEFGTVNEKLFF	TRBJ1-4	TRBC1	1.73%
TRAV14/DV4	CAMREVNTGNQFYF	TRAJ49	TRAC	TRBV19	CASSGLAAPYNEQFF	TRBJ2-1	TRBC2	1.10%
TRAV8-6	CAVLRLSF	TRAJ20	TRAC	TRBV9	CASSVEGGYYNEQFF	TRBJ2-1	TRBC2	1.07%

**Table 3:** Top 10 TCR clonotypes information of representative sample from Discharged group (Discharged#2).

TRAV	TRA-CDR3	TRAJ	TRAC	TRBV	TRB-CDR3	TRBJ	TRBC	Ratio
TRAV26-1	CIVRSPTGDSWGKLQF	TRAJ24	TRAC	TRBV4-1	CASSQDRGNMNTEAFF	TRBJ1-1	TRBC1	0.44%
TRAV8-3	CAVGAKGYQKVTF	TRAJ13	TRAC	TRBV27	CASSLSNPRDEQFF	TRBJ2-1	TRBC2	0.37%
TRAV27	CAGHNAGNNRKLIW	TRAJ38	TRAC	TRBV4-1	CASSQGLAGANEQFF	TRBJ2-1	TRBC2	0.29%
TRAV16	CALSRGSNYKLTF	TRAJ53	TRAC	TRBV5-6	CASSPWRLDSLWGGYTF	TRBJ1-2	TRBC1	0.18%
TRAV27	CAGAKGNNDMRF	TRAJ43	TRAC	TRBV13	CASSFQGRGTEAFF	TRBJ1-1	TRBC1	0.18%
TRAV3	CADYYGQNFVF	TRAJ26	TRAC	TRBV28	CASSFQGFTEAFF	TRBJ1-1	TRBC1	0.17%
TRAV1-2	CAVWDSNYQLIW	TRAJ33	TRAC	TRBV6-2	CASSYGGDTGELFF	TRBJ2-2	TRBC2	0.15%
TRAV5	CAESIRRDKIIF	TRAJ30	TRAC	TRBV4-1	CASSWDPTGNTEAFF	TRBJ1-1	TRBC1	0.15%
TRAV20	CAVLSGAGSYQLTF	TRAJ28	TRAC	TRBV9	CASSVESGTGWGKLFF	TRBJ1-4	TRBC1	0.13%
TRAV10	CVVSGGGADGLTF	TRAJ45	TRAC	TRBV29-1	CSGTGANSYEQYF	TRBJ2-7	TRBC2	0.11%

**Table 4:** Top 10 TCR clonotypes information of representative sample from Follow-up group (Follow-up#3).

TRAV	TRA-CDR3	TRAJ	TRAC	TRBV	TRB-CDR3	TRBJ	TRBC	Ratio
TRAV1-2	CAVEDSNYQLIW	TRAJ33	TRAC	TRBV19	CASDGTGGSGANVLTF	TRBJ2-6	TRBC2	4.16%
TRAV29/DV5	CAASADFNKFYF	TRAJ21	TRAC	TRBV27	CASSPRTGEIAKNIQYF	TRBJ2-4	TRBC2	3.34%
TRAV13-1	CAASYFGNEKLTF	TRAJ48	TRAC	TRBV7-9	CASSSPRGRNEQFF	TRBJ2-1	TRBC2	2.70%
TRAV12-2	CAVNVGGAALIF	TRAJ15	TRAC	TRBV4-3	CASSQDGAGADEQFF	TRBJ2-1	TRBC2	2.68%
TRAV22	CAVEPPSGTYKYIF	TRAJ40	TRAC	TRBV5-5	CASSSLEGEETQYF	TRBJ2-5	TRBC2	2.36%
TRAV1-2	CAWQAGTALIF	TRAJ15	TRAC	TRBV5-1	CASSSVGGNEQFF	TRBJ2-1	TRBC2	2.01%
TRAV19	CALSEASNYGQNFVF	TRAJ26	TRAC	TRBV2	CASRSGTSDHEQYF	TRBJ2-7	TRBC2	1.45%
TRAV17	CATSLIQGAQKLVF	TRAJ54	TRAC	TRBV4-1	CASSHANTGELFF	TRBJ2-2	TRBC2	1.25%
TRAV17	CATDEGGSYIPTF	TRAJ6	TRAC	TRBV28	CASSFPSGARGYTF	TRBJ1-2	TRBC1	1.08%
TRAV13-1	CAGDTGRRALTF	TRAJ5	TRAC	TRBV5-1	CASSGDRGPGTEAFF	TRBJ1-1	TRBC1	0.73%

### Differentially expressed genes related to immune functions in recovered patients

We next focused on the expanded top T cell clones, representing the most active T cells responding against SARS-CoV-2 infection. We linked scRNA-seq and paired scTCR-seq data from the HD group (17,374 cells), the Discharged group (24,040 cells) and the Follow-up group (24,938 cells) (Supplementary Table S4). In each group, the cell number of the top 20 TCR clonotypes contributed ~10% to 20% of total cell number (Supplementary Table S4). We first explored *CD3E*, *CD4*, and *CD8A* expression in the TCR paired scRNA-seq data to identify *CD3E*^+^*CD8A*^+^*CD4*^*−*^ populations. The integrated data from HD *vs*. Discharged *vs*. Follow-up groups showed a comparable cell number of *CD3E*^+^*CD8A*^+^*CD4*^*−*^ cluster with that of *CD3E*^+^*CD8A*^*−*^*CD4*^+^ cluster (Supplementary Fig. S4). Whereas, the *CD3E*^+^*CD8A*^+^*CD4*^*−*^ clusters contributed substantially to all of top 20 TCR data in three groups’ integrated data (Supplementary Fig. S5, Table S4), which indicated expanded CD8^+^ T cell clones contributing to immune response to viral infection.

**Fig. 4:**
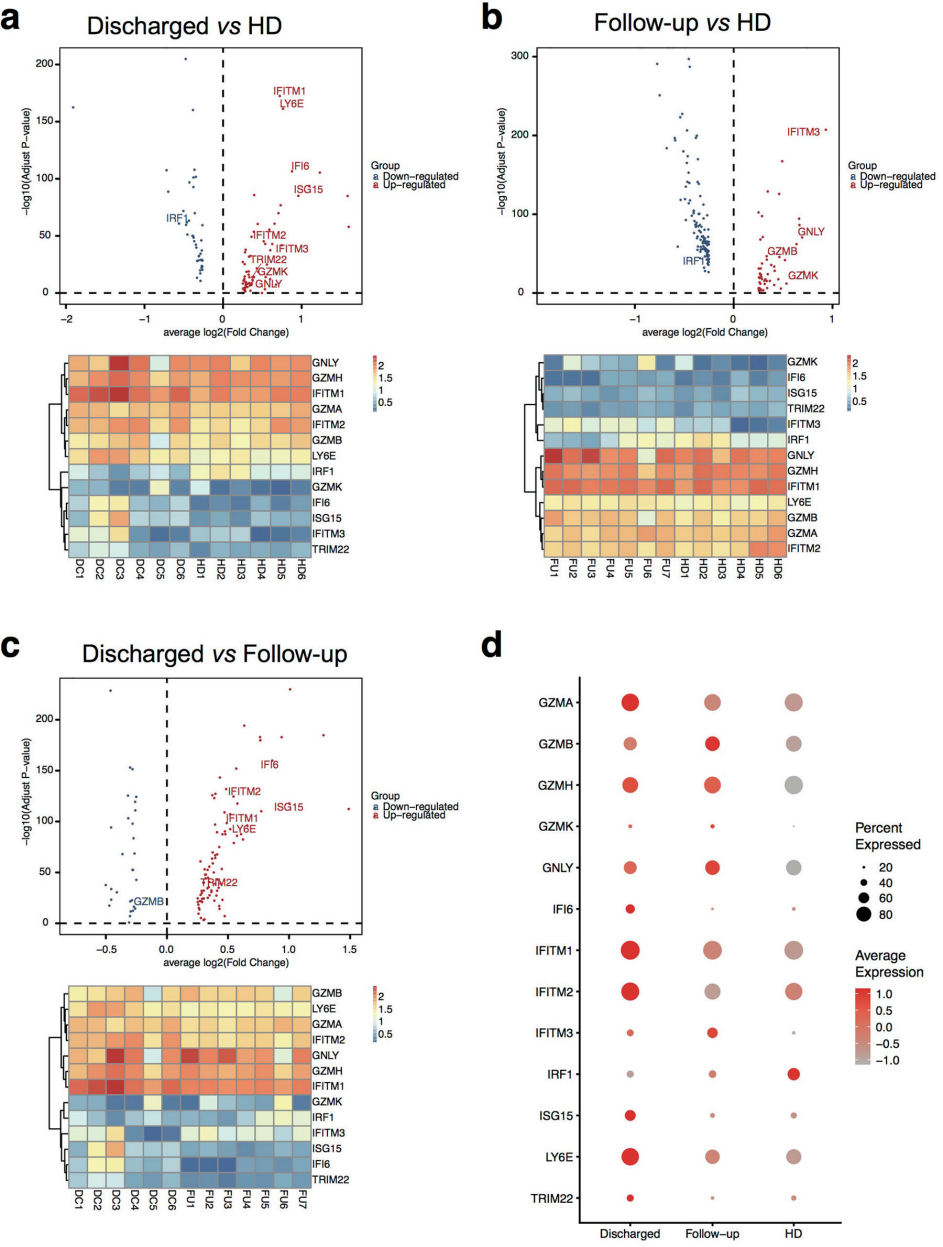
Deferentially expressed immune-related genes of CD8^+^ subset from top 20 TCR clonotypes paired scRNA-seq data. The volcano plot of significantly changed genes and the heatmap of selected granzyme- and interferon-related genes of *CD3E*^+^*CD8A*^+^*CD4*^−^ clusters in top 20 TCR clonotypes from Discharged group *vs*. HD group (*n* = 6 per group) (**a**), from Follow-up group *vs*. HD group (*n* = 7 in Follow-up group, *n* = 6 in HD group) (**b**), and from Discharged group *vs*. Follow-up group (**c**). **d** The bubble plot of selected granzyme- and interferon-related genes’ expression level in Discharged, Follow-up, and HD groups (*n* = 6 in Discharged and HD group, *n* = 7 in Follow-up group) of *CD3E*^+^*CD8A*^+^*CD4*^−^ clusters in top 20 TCR clonotypes. DC, Discharged group; FU, Follow-up group; HD, Healthy Donor group.

By comparing the differentially expressed genes from top 20 TCR clonotypes within *CD3E*^+^*CD8A*^+^*CD4*^−^ clusters in healthy donors and recovered patients, we found that both the Discharged and Follow-up groups expressed an increased fold change of IFN and granzyme-related genes expression compared with healthy donors (Fig. 4a, b). In comparison with the HD group, four IFN-related genes (*IFITM1*, *IFITM2*, *IFITM3*, and *IFI6*) and IFN activation downstream related genes (*ISG15* and *TRIM22*) increased in the Discharged group, indicating an enhanced anti-viral immunity in the Discharged group (Fig. 4a). Moreover, the highly expressed cytotoxic genes (*GZMK* and *GNLY*) also demonstrated that the T cell clones from the Discharged group possessed a powerful killing function against the SARS-CoV-2 infected cells. In Follow-up *vs*. HD analysis, the Follow-up group exhibited similar highly expressed IFN-related gene *IFITM3* and granzyme/perforin-related genes (*GZMB*, *GZMK*, and *GNLY*) (Fig. 4b). Thus, the anti-viral immunity and the cytotoxicity persisted at a high level in the recovered patients at both Discharged and Follow-up time points.

Interestingly, we found the key IFN regulator gene *IRF1* downregulated in both Follow-up and Discharged groups, which may contribute to the restriction of viral replication in the T cells of SARS-Cov-2 infected patients (Fig. 4a, b). Furthermore, with a direct comparison between the Discharged group and the Follow-up group, we found a significantly higher expression level of CD8^+^ T cell anti-viral functional genes in the Discharged group, most of which were the IFN-related genes (*IFITM1*, *IFITM2*, *IFI6*, *TRIM22*, and *ISG15*) (Fig. 4c). Previous studies indicated that IFITM proteins were employed in the SARS infection for restricting virus fusion^19,20^. The recent reported LY6E, which impairs the coronavirus fusion and inhibits the SARS-CoV-2 infection^21^, was also highly expressed in the Discharged group. In addition, a mixed expression pattern of cytotoxic genes was observed, i.e., *GZMA* and *GZMH* were highly expressed in the Discharged group, whereas *GZMB* and *GNLY* showed an increased expression in the Follow-up group (Fig. 4d).

Overall, the highly expressed *IFITM* family and *LY6E* genes in the Discharged group implied a better defense against SARS-CoV-2 infection by the resistance of target cells to viral fusion than other two groups. Both Discharged and Follow-up groups maintained a powerful viral elimination competence with a strong killing function. These data suggested, along with the recovery from SARS-CoV-2 infection, that the anti-viral fusion potency would revert to the basal level quickly, while the killing features of CD8^+^ T cells persist for a longer time.

### IFN-related anti-viral features in the discharged patients

**Fig. 5:**
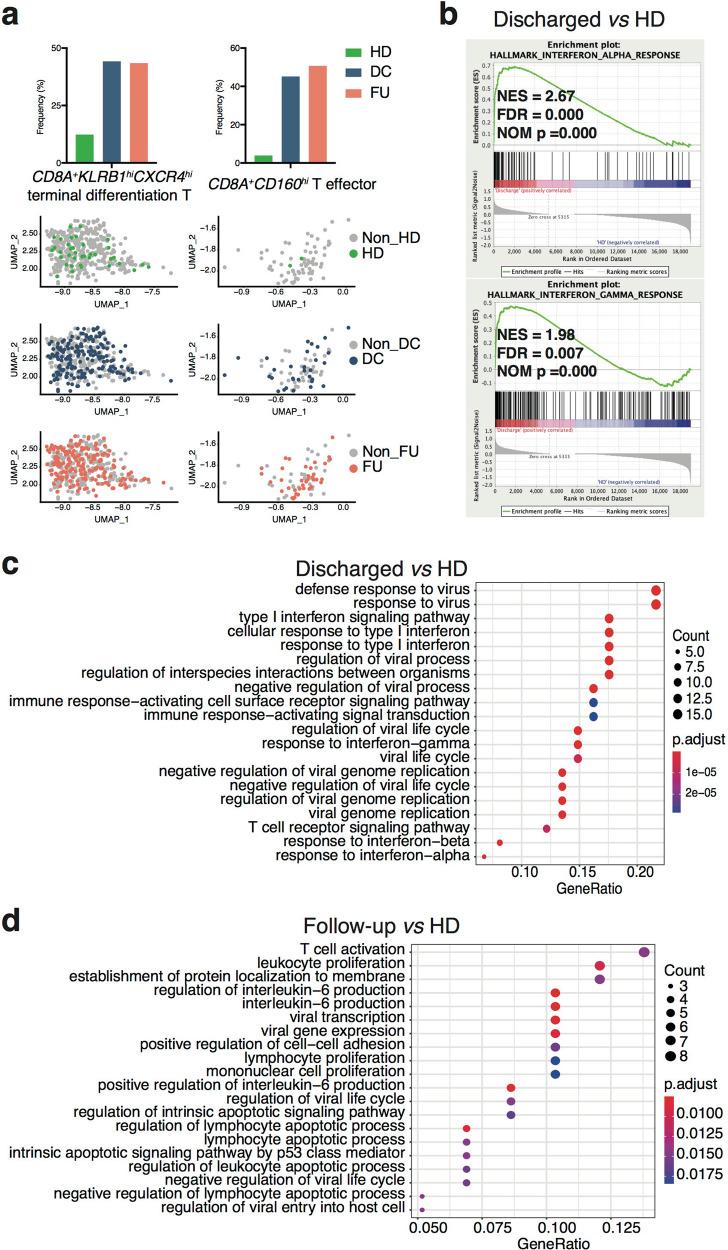
GSEA and biological process analysis for *CD3E*^+^*CD8A*^+^*CD4*^*−*^ clusters of top 20 TCR clonotypes paired scRNA-seq data from Patient group *vs*. HD group. **a** Distribution of each group in *CD8A*^+^*KLRB1*^*+*^*CXCR4*^+^ terminal differentiation T subset (left panel) and *CD8A*^+^*CD160*^*hi*^ effector T subset (right panel). **b** Using GSEA to analyze expressed genes from *CD3E*^+^*CD8A*^+^*CD4*^−^ clusters of top 20 TCR paired scRNA-seq data, 2 gene sets of interferon response significantly upregulated in Discharged group comparing with HD group (*n* = 6 per group). NES, normalized ES; FDR, false discovery rate; NOM p, normalized *p* value. Top 20 BP enrichment analysis of DEGs from *CD3E*^+^*CD8A*^+^*CD4*^−^ clusters of top 20 TCR paired scRNA-seq data, which were upregulated in Discharged group *vs*. HD group analysis (*n* = 6 per group) (**c**) and Follow-up *vs*. HD groups (*n* = 7 in Follow-up group, *n* = 6 in HD group) (**d**). DC, Discharged group; FU, Follow-up group; HD, Healthy Donor group.

The features of immune genes in CD8^+^ T cells from top 20 clonal T cells indicated their pivotal effector function in the anti-viral immune responses. We next illustrated the significant properties of these expanded CD8^+^ T subsets in COVID-19 recovered patients by Gene Set Enrichment Analysis (GSEA) with expressed genes in the Discharge group *vs*. the HD group. Via gene expression in each cluster, we identified the subsets of top 20 clonal T cells (Supplementary Figs. S7–S9). Interestingly, the Discharged and Follow-up groups contributed the majority of *CD8A*^+^*KLRB1*^*hi*^*CXCR4*^*hi*^ terminal differentiation T subset and *CD8A*^+^*CD160*^*hi*^ T effector subset (Fig. 5a), in which the Discharged group revealed anti-viral hallmarks by GSEA contrast to HD group (Fig. 5b). Furthermore, we analyzed biological process (BP) based on Gene Ontology (GO) database^22,23^ of differentially expressed genes of CD8^+^ T subsets in top 20 clonal T cells. Comparing with the HD group, the patients displayed anti-viral immune response BPs (Fig. 5c, d). Particularly in the Discharged group, most of the top 5 BPs (3/5) directly linked to interferon-related anti-viral immune response (Fig. 5c).

Moreover, the gene concept network analysis of differentially expressed genes also supported the pathway analysis of Discharged group (Supplementary Fig. S10). Consistently, the expressed genes of total CD8^+^ T cells in the Discharged group displayed exactly the same two interferon-related hallmarks compared to the HD group based on GSEA (Supplementary Fig. S11a). Moreover, total CD8^+^ T cells also exhibited 8 anti-viral BPs out of the top 10 BPs (Supplementary Fig. S11b).

Taken together, the GSEA and BP results of CD8^+^ T cells from top 20 clones were remarkably similar to the analysis results from total CD8^+^ T cell population, although top 20 TCR clonotypes contributed <20% of the total T cells. These data indicate top 20 T cell clones were the active populations representative of IFN-dependent anti-viral functional features at the discharge stage.

### Alteration of metabolism in the Follow-up patients

**Fig. 6:**
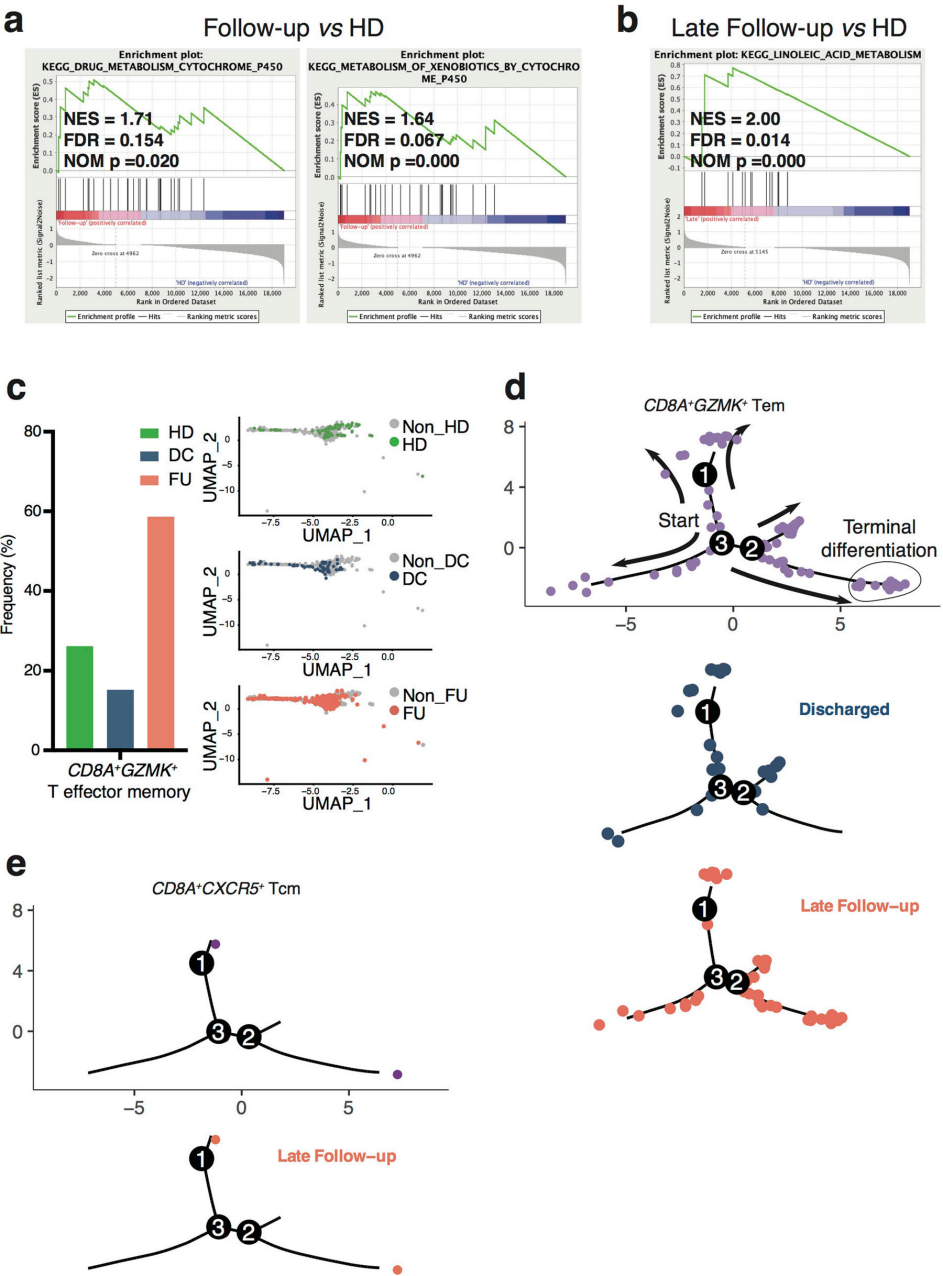
GSEA for *CD3E*^+^*CD8A*^+^*CD4*^−^ clusters of top 20 TCR clonotypes paired scRNA-seq data from Follow-up group *vs*. HD group and pseudo-time analysis of paired samples from Discharged and Late Follow-up groups. **a** Using GSEA to analyze expressed genes from *CD3E*^+^*CD8A*^+^*CD4*^−^ clusters of top 20 TCR paired scRNA-seq data, 2 metabolism-related gene sets significantly upregulated in Follow-up group comparing with HD group (*n* = 7 in Follow-up group, *n* = 6 in HD group), and **b** a fatty acid metabolic gene set significantly upregulated in Late Follow-up group *vs*. HD group analysis (*n* = 4 in Late Follow-up group, *n* = 6 in HD group). NES, normalized ES; FDR, false discovery rate; NOM p, normalized *p* value. **c** Distribution of each group in *CD8A*^+^*GZMK*^+^ T effector memory subset. **d** Pseudo-time analysis of top 20 T cell clones from Discharged and Late Follow-up paired samples. Upper panel, *CD8A*^+^*GZMK*^+^ Tem subset of combined Discharged and Late Follow-up samples. The arrows indicate the cells’ differentiation trajectories from the beginning to the end; Lower panel, separated Discharged and Late Follow-up groups in *CD8A*^+^*GZMK*^+^ Tem subset (*n* = 3 per group). **e** Pseudo-time analysis of top 20 T cell clones from Discharged and Late Follow-up paired samples. Left panel, distribution of *CD8A*^+^*CXCR5*^+^ Tcm subset; right panel, only the Late Follow-up samples contributed in *CD8A*^+^*CXCR5*^+^ Tcm subset (*n* = 3 per group). DC, Discharged group; FU, Follow-up group; HD, Healthy Donor group; Tcm, central memory T cells; Tem, effector memory T cells.

Top 20 clones of CD8^+^ T cells in the Follow-up group displayed 2 significantly enriched gene sets about metabolism comparing with the HD group, and none of the viral infection-related gene sets was identified with GESA (Fig. 6a). The GSEA result based on total CD8^+^ T cell indicated the same metabolic features in the Follow-up group (Supplementary Fig. S12).

We next divided the Follow-up group into Early and Late subgroups based on their sampling time points (Fig. 1) (Supplementary Table S1). By direct comparison with the HD group in top 20 clones of CD8^+^ T cells, the Early Follow-up group displayed strong metabolism features: all of the top 3 BPs of differentially expressed genes were related to metabolism (Supplementary Fig. S13). While in contrast to the HD group, the Late Follow-up group showed fatty acid metabolism feature specifically by GSEA (Fig. 6b). Previous studies indicated that the memory T cells increased fatty acid oxidation with more spare respiratory capacity for rapidly recall upon challenge^24,25^. In the top20 clones’ data, we have identified two memory T subsets: central memory T subset and effector memory T subset (Supplementary Fig. S7). We focused on analyzing *CD8A*^+^*GZMK*^+^ effector memory T subset, of which cells could rapidly differentiate into effector cells against the infection^26^. We found that main constituent of this effector memory T subset was from the Follow-up group (Fig. 6c). The transcription factor analysis indicated that the Late Follow-up group displayed specifically active transcription factor SP1, linked to lipid metabolism and cholesterol biosynthesis^27–29^, in *CD8A*^+^*GZMK*^+^ effector memory T subset (Supplementary Fig. S14).

Via Monocle analysis of paired Discharged and Late Follow-up samples in top 20 TCRs paired scRNA-seq data, we found that the Late Follow-up group contributed a larger proportion in *CD8A*^+^*GZMK*^+^ effector memory T cell population, and distributed along the terminal-exhausted differentiation. While the Discharged group located relatively close to the beginning of trajectories (Fig. 6d). Moreover, the Monocle analysis showed that the Late Follow-up group contributed all cells in *CD8A*^+^*CXCR5*^+^ central memory T subset (Fig. 6e). These data demonstrated that the Follow-up group in top 20 CD8^+^ T cell clones displayed multiple metabolic features. In particular, the Late Follow-up group exhibited remarkable memory T cells metabolic features, coinciding with CD8^+^ T cells differentiation during anti-viral immune response.

Overall, we assumed that the patients possessed a higher proficiency against SARS-CoV-2 infection during recovery stages, and vital process was changed from anti-viral immune response to metabolism adaptation after discharge.

## Discussion

This study used a combination of scTCR-seq and scRNA-seq analysis to measure both TCR clone dynamics and functional gene expression in COVID-19 convalescent individuals. Three key findings are shown from our systemic analysis: (1) TCR repertoire diversity decreased quickly after recovering from COVID-19, without changing the global frequency of VDJ gene usage; (2) The dynamics of TCR repertoire was linked to a profound change of gene signatures from anti-viral response to metabolism adaptation; (3) The top expanded T cell clones (~10% of total T cells) determined the principal features of CD8^+^ T cells, indicating the vital role of antigen-specific T cells in fighting against SARS-CoV-2 infection.

**Fig. 7:**
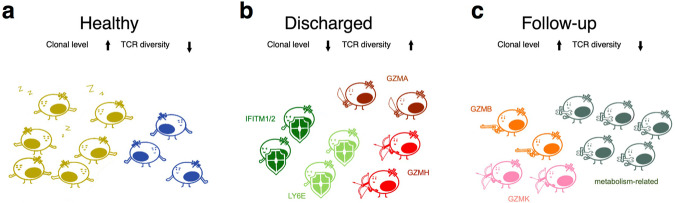
A model for CD8^+^ T cell immune dynamics in recovered COVID-19 patients. **a** The minor clones of CD8^+^ T cells in HD group were the immune surveillance cells with high clonal expansion level and low TCR diversity. **b** CD8^+^ T cells of Discharged group were minor clones with low clonal expansion but high TCR diversity, which displayed powerful defending and cytotoxicity effects via interferon- and granzyme-related genes’ expression. **c** In the Follow-up group, CD8^+^ T cells remained a few minor clones of cytotoxicity and large clones with active metabolic reprogramming. Different colors of T cells represent they are from different clones. Weapons and shields on T cells represent that T cells express functional genes for killing the infected cell and defending themselves from SARS-CoV-2 infection, respectively.

Our study demonstrated exciting features of T cell responses during COVID-19 recovery process. A previous study provided evidence that SARS-CoV-2 infection resulted in T cell reduction due to the functional impairment of dendritic cells, and these weakened/delayed CD8^+^ T cell responses could contribute to acute COVID-19 pathogenesis^30^. Our data indicated a low level of clonal expansion CD8^+^ T cells, with a strong anti-viral function (both defending and killing effects), which could serve as the major players in combating the virus before or during discharge time point (Fig. 7b). Our findings, at least partially, solved a conundrum: How a dramatically decreased level of CD8^+^ T cell can clear the virus in most SARS-CoV-2 infected individuals.

The granzyme favors the cytolysis of viral infected cells with different properties^31,32^. GZMA, GZMB, and GZMK act as pro-inflammatory factors^33–35^, of which GZMB is the most powerful pro-apoptotic granzyme^36^, and *GNLY* encodes granulysin protein binding to cells via electric charge to lysis target cells^37^. Furthermore, the studies in mice suggested that GZMA and GZMB possess distinct cytotoxic sensitivity to different target cells via perforin-mediated apoptosis^36,38^. The Discharged group showed high expression of *GZMA* (Fig. 4d), which had relatively low cytotoxicity^36^. While *GZMB*, a greater cytotoxic granzyme^36^, was highly expressed in the Follow-up group. The cytotoxic genes expression indicated that viral clearance was not the end of the immune response, and afterward, immune system preserved cytotoxicity in a period with different characteristics. Based on the distinct expression of granzymes in different groups, we assume that: immune system eliminates infected cells and viruses via a cytotoxic mediator network and is a manifold system; the cytotoxic function is enlarged with pro-apoptotic factors such as GZMB and GNLY over time; in late-stage afterward viral clearance, immune system maintains pro-apoptotic features, which could efficiently avoid repeat infection in the short term.

IRF1 is a key regulator that contributes to anti-viral response. Previous studies indicated that IRF1 polymorphisms (down-regulation) were associated with RNA virus HIV-1, infection resistance^39^ and modulated by HIV-1 to enhance viral replication and penetrated immune system defense at least at the initial stage^40^. Our data revealed that during SARS-CoV-2 infection, a single-strand RNA virus, CD8^+^ T cells exhibited low *IRF1* and high *IFITM* levels in the Discharged and Follow-up groups. Thus, we hypothesized that immune system probably used a similar mode against HIV-1 infection by down-regulated *IRF1* and up-regulated *IFITM* to avert the direct viral infection in CD8^+^ T cells.

In addition, we found 1–6 weeks after discharge (follow-up time point), the clonal expansion increased, and the diversity of TCR repertoire decreased to basal level, comparable with healthy donors, which were not exposed to SARS-CoV-2. Moreover, the clonally expanded CD8^+^ T cells in the Follow-up group exhibited features of active metabolic reprogramming, supporting a post-infection recovery for the T cell system (Fig. 7c). In addition, the Early Follow-up group showed the ATP metabolism feature in biological process analysis, which corresponded to the substantial spare respiratory capacity of long-lived CD8 memory T cells after clearance of infection to produce sufficient ATP and promote CD8 memory T cells survival^41,42^. Previous studies suggested that upregulated carnitine palmitoyl transferase-I (the first step in fatty acid oxidation) in CD8 memory T cells increased fatty acid oxidation and mitochondrial respiratory capacity^24^. Our results indicated that in the late Follow-up group, fatty acid metabolism increased significantly. Therefore, immune system could quickly respond to viral challenge.

Overall, in the Healthy group, the minor clones were immune surveillance cells and maintained homeostasis of immune response (Fig. 7a); in the Discharged group, most low expanded minor clones were defensing against virus infection (IFN-related) and cytotoxic (granzyme) clones (Fig. 7b); and the Follow-up group possessed GZMB-cytotoxic clones and the major metabolic clones (Fig. 7c). Our data indicated a low level of clonal expanded CD8^+^ T cells, with a strong anti-viral function, which could serve as the pivotal factor during discharged time point. The clonal expansion increased, and the diversity of TCR repertoire decreased to basal level as the healthy donors within 6 weeks after discharge. Furthermore, the clonally expanded CD8^+^ T cells in the Follow-up group exhibited features of active metabolic reprogramming, revealing a potential post-infection recovery for T cell immune system.

We believe that investigating the dynamics of TCR repertoire could provide a valuable tool to monitor T-cell-mediated immune responses to potential SARS-CoV-2 vaccines. This area of research will need to be expanded using a larger cohort of patients, which could be sampled at different time points to provide more detailed information in near future for designing the best way to increase the number of T cells and enhance their anti-viral function. Moreover, our presented study could form the basis for developing a novel T cell vaccine to combat SARS-CoV-2 infection.

## Materials and methods

### Key resources table

**Table:** 

Reagent or resource	Source	Identifier
Chemicals		
Ficoll-Paque	GE Healthcare	Cat# 17-1440-02

### Experimental model and subject details

#### Samples collection

The peripheral blood samples were collected from 10 patients and 6 Healthy Donors (HD). Six patients, sampling on the discharged day or within 1 week before discharge, were identified as Discharged group (DC). The samples of the Follow-up group (FU) were collected during 1 week to 6 weeks after discharge. The Follow-up group was divided into the Early Follow-up group (EFU) and the Late Follow-up group (LFU) according to the days from discharge. Briefly, 3 patients of the Early Follow-up group were sampled on 7 days post-discharge. The Late Follow-up group contained 4 patients, in which 3 patients from the Discharged group were re-sampled between 19 and 40 days post-discharge and 1 patient was sampled on 30 days post-discharge (Fig. 1a). Detailed patient and grouping information were provided in Supplementary Table S1. This study was under the tenets of the Declaration of Helsinki and was approved by the Medical Ethical Committee at Shanghai Jiao Tong University School of Medicine, China.

### Method details

#### PBMC isolation, single-cell RNA sequencing, and analysis

For the single-cell RNA and TCR sequencing, isolated PBMCs were prepared by the standard density gradient centrifugation with Ficoll-Paque (GE Healthcare, Uppsala, Sweden). Prepared PBMCs were used to construct scRNA-seq libraries. The Chromium Single-Cell 5’ v3 Reagent Kit (10X Genomics, Pleasanton, USA) was used as per the manufacturer’s instructions. The Cell Ranger software (version 3.1.0) was used to process the single-cell expression data and align data to the GRCH38 reference genome, of which the corresponding vdj package was used for TCR v/d/j analysis (Supplementary Table S2). The gene expression matrixes of samples were converted to Seurat objects via Seurat (v3.2.0, R package)^43^ in R (v3.6.0). According to samples’ variation, the following criteria were applied to each sample: mitochondrial gene percentage <5%; gene number more than 200 and less than 2200 (for #DC3), 2500 (for #HD1/2/3), 3000 (for #DC2/4/5/6 and samples from Follow-up group), 4000 (for #DC1 and #HD4/5), and 4500 (for #HD6). Furthermore, the cells, expressing all of TCR α chains, TCR β chains and transcriptional RNA data, were kept. After filtering, 66352 cells (2740/2323/2239/3852/2744/3476 for HD samples; 5237/5446/4483/2034/3412/3428 for DC samples; 4824/2419/3419/4689/2859/3795/2933 for FU samples) were retained for following analysis (Supplementary Tables S3 and S4). The gene expression matrixes were normalized and calculated with the top 4000 variable genes by ‘NormalizeData’ and ‘FindVariableFeatures’ functions in the Seurat package. The gene expression matrixes were integrated together using ‘FindIntegrationAnchors’ and ‘IntegrateData’ functions with the 50 dimensions from CCA to correct the batch effect from samples (Supplementary Fig. S3). The integrated data were saved for following analysis. We scaled the integrated data with default parameters in ‘ScaleData’ function. The 50 principal components were used in the principal component analysis and followed by UMAP. We applied top 20 principal components for ‘FindNeighbors’ and the following ‘FindClusters’ functions for clustering. Moreover, data were saved for the following differentially expressed genes (DEGs) analysis. To visualizing *CD3E*, *CD4*, and *CD8A* genes expression, ‘FeaturePlot’, ‘VlnPlot’, and ‘CombinePlot’ functions were performed. CD4^+^ and CD8^+^ clusters were identified by *CD3E*, *CD4*, and *CD8A* genes expression. CD8^+^ clusters from integrated data were sorted with *CD3E*^+^*CD4*^*−*^*CD8A*^+^ genes expression via the ‘subset’ function in Seurat and saved for following GSEA analysis.

### Top 20 TCR clonotypes *CD3E*^+^*CD4*^*−*^*CD8A*^+^ cluster analysis

Using Cell Ranger vdj package, the V/D/J and CDR3 sequences of TCR α and β chains were paired by the barcode and ranked the TCR clonotypes by the abundance. The cells, which expressed top 20 TCR clonotypes in each sample, were analyzed with the same approach of Seurat. Briefly, the cells expressed top 20 TCR clonotypes in previous created integrated data were selected and applied ‘ScaleData’, ‘RunPCA’, ‘RunUMAP’, ‘FindNeighbors’, and ‘FindClusters’ functions with the same parameters as mentioned before. The data were saved for subsequent differentially expressed genes analysis and gene set enrichment analysis. Moreover, we applied ‘FeaturePlot’, ‘VlnPlot’, ‘CombinePlot’, and ‘DotPlot’ functions for identifying CD4^+^ and CD8^+^ clusters by gene markers’ expression. CD8^+^ clusters from top 20 TCR clonotypes paired integrated data were sorted with *CD3E*^+^*CD4*^−^*CD8A*^+^ genes expression via the ‘subset’ function in Seurat. Then, the functional gene expression in CD8^+^ T cell clusters was visualized by ‘FeaturePlot’ function in Seurat. In CD8^+^ clusters from integrated data, CD8^+^ T cell functional genes were performed using ‘DotPlot’ function with ‘dot.scale’ as 8.

### Cell type annotation

The corresponding cell types of the clusters were annotated manually in accordance with known gene markers (Supplementary Figs. S6, S9)^44,45^.

### Differential expression analysis and visualization

The differentially expressed genes in CD4^+^ subset, and CD8^+^ subset were identified using ‘FindMarkers’ function with subset.ident parameter as *CD3E*^+^*CD4*^+^*CD8A*^−^ and *CD3E*^+^*CD4*^*−*^*CD8A*^+^ clusters, respectively, in above-mentioned data. Moreover, we compared the Discharged group to the HD group, the Follow-up group to the HD group, and the Discharged group to the Follow-up group differential expressed genes in CD4^+^ and CD8^+^ subsets by ident.1 parameter. The differentially expressed genes were up-regulated and down-regulated according to the avg_logFC value >0 and <0. For visualizing the genes differential expression, we applied ‘ggscatter’ function in ggplot2 (v3.3.2, R package) with ‘size’ as 1 and true of ‘repel’, the default setting ‘theme_base’ function in ggthemes (v4.2.0, R package) and the default setting ‘geom_hline’ and ‘geom_vline’ functions in ggpubr (v0.4.0, R package).

### TCR sequencing analysis

TCR sequencing data, which displayed CDR3 sequences, were kept for TCR α and β chains analysis. ‘as.character’, ‘nchar’, ‘unlist’, ‘table’, and ‘round’ functions were used in TCR α and β chains’ CDR3 length and V/J distribution analysis. The productive cells with CDR3 sequences and C genes, which expressed both TCR α and β chains, were sorted for the clonal expansion, V/CDR3/J usage in top 10 TCR clonotypes and V–J pairing analysis of every sample. Briefly, TCR α and β chains were separated, and the duplicated barcodes of each chain were removed. ‘as.data.frame’, ‘unlist’, ‘table’, ‘round’, ‘order’, and ‘cumsum’ functions were used in clonal expansion analysis. Furthermore, we visualized the analysis result with ‘ggplot’, ‘geom_rect’, ‘coord_polar’, ‘labs’, ‘xlim’, ‘theme_light’, ‘theme’, and ‘scale_fill_manual’ functions in ggplot2 package. For V/CDR3/J usage in top 10 TCR clonotypes, we applied ‘str_split_fixed’ function in stringr (v1.4.0, R package), ‘paste’, ‘as.data.frame’, ‘table’, and ‘order’ functions; V/CDR3/J usage in top 10 TCR clonotypes was visualized by ‘ggplot’, ‘scale_x_discrete’, ‘theme_minimal’, ‘theme’, ‘ggtitle’, ‘scale_fill_discrete’, ‘geom-alluvium’, and ‘geom_stratum’ functions in ggplot2 and ggalluvial (v0.12.0, R package). In addition, the ‘str_split_fixed’ function in stringr, ‘as.data.frame’, ‘paste’, and ‘table’ functions were employed for V–J pairing analysis; we applied ‘chordDiagram’ and ‘circos.trackPlotRegion’ functions in circlize (v0.4.10, R package) for V/J pairing visualization.

### Biological process enrichment and gene concept network analysis

The upregulated differentially expressed genes, which were from previous ‘FindMarkers’ function analysis, were applied ‘bitr’ function and ‘enrichGO’ function from clusterProfiler (v3.14.3, R package)^46^ with org.Hs.eg.db (v3.11.4, R package)^47^ for ‘OrgDb’ parameter. For setting ‘enrichGO’ function, BP was as ‘ont’ with 0.05 for ‘pvalueCutoff’ and ‘qvalueCutoff’. BP pathways were displayed using ‘dotplot’ function with 20 for ‘showCategory’ parameter in enrichplot (v1.6.1, R package). Furthermore, the gene-concept networks of top 5 BP pathways were visualized by ‘cnetplot’ in enrichplot (v1.6.1, R package).

### Gene set enrichment analysis

The data from previous Seurat applications were employed in gene set enrichment analysis (GSEA). We added the group information in the data and got the gene expression matrix by ‘GetAssayData’ function in Seurat with ‘slot’ parameter as data. The gene expression matrix of each sample was sorted, and the mean value of every gene was calculated by ‘rowMeans’ function. The gene expression matrix file (“.gct” format) and phenotype file (“.cls” format) of samples were loaded into GSEA (v4.1.0). Then, GSEA was performed ‘h.all.v7.1.sumbols.gmt [Hallmarks]’ and ‘c2.cp.kegg.v7.1.symbols.gmt [Curated]’ gene sets database with 2000 permutations, ‘No_Collapse’ and Permutation type as ‘gene-set’.

### Single-cell development trajectory reconstruction

The Monocle2 (v2.14.0, R package) was applied to construct single-cell trajectories to discover developmental transitions. The clustering top 20 TCR clonotypes scRNA-seq data from paired samples in Discharged, and Late Follow-up group were performed the pseudo-time analysis. Briefly, genes in which the count of cells expressing is greater than or equal to 50, were used to define cells’ progress and clustering in ‘differentialGeneTest’ and ‘setOrderingFolter’ functions. ‘DDRTree’ was applied in the ‘reduceDimension’ function to reduce dimensions, and ‘plot_cell_trajectory’ function was used for visualization.

### Transcription factor analysis

The top 20 clones’ gene expression matrixes and annotation information of each group were uploaded to IRIS3^48^ website (https://bmbl.bmi.osumc.edu/iris3/submit.php) for transcription factor analysis. The regulon analysis of each group was performed with Job ID 20210620233403 for late Follow-up group, Job ID 20210620212155 for early Follow-up group, Job ID 20210620190351 for Discharged group, Job ID 2021062104159 for the HD group.

### Quantification and statistical analysis

#### Disease severity quantification

We quantified illness severity by a given criterion, which was proposed by WHO committee Ordinal Scale (WOS)^49^: (0) uninfected—no evidence of infection; (1) ambulatory—no limitation of activities; (2) ambulatory—limitation of activities; (3) hospitalized mild disease—hospitalized without oxygen therapy; (4) hospitalized mild disease—hospitalized with oxygen by mask or nasal prongs; (5) hospitalized severe disease—non-invasive ventilation of high-flow oxygen; (6) hospitalized severe disease—intubation and mechanical ventilation; (7) hospitalized severe disease—ventilation + additional organ support—pressors, RRT, ECMO; (8) dead—death.

#### TCR diversity calculation

The ratio of TCR classification count to total cell number in each sample was used to measure the clonotype diversity of samples. Briefly, we defined the number of different TCR sequences as the TCR classification count, and the percentage of the sample’s clone diversity = (TCR classification count/total cell count) × 100%. Thus, the sample displayed a low ratio that means the sample possessed either decreased TCR classification count or increased total cell number, of which the TCR diversity is lower.

### Statistical analysis

The statistical analysis was performed by Prism 7 software with unpaired student’s *t*-test in the two-group analysis. *P values < 0.05, **P values < 0.01, ***P values < 0.001.

## Supplementary information

Supplementary Information

## Data Availability

The raw sequence data reported in this paper have been deposited in the Genome Sequence Archive of the Beijing Institute of Genomics (BIG) Data Center, BIG, Chinese Academy of Sciences, under accession code HRA000297 and PRJCA004747, which is publicly accessible at http://bigd.big.ac.cn/gsa-human/.

## Materials availability

All unique reagents and materials generated in this study are available from the Lead Contact (Feng Wang, wangfeng16@sjtu.edu.cn) upon request.

## References

[1] Guan WJ (2020). Clinical characteristics of coronavirus disease 2019 in China. N. Engl. J. Med..

[2] WHO. *World Health Organization. Statement on the second meeting of the International Health Regulations (2005) Emergency Committee regarding the outbreak of novel coronavirus (2019-nCoV)*, https://www.who.int/news-room/detail/30-01-2020-statement-on-the-second-meeting-of-the-international-health-regulations-(2005)-emergency-committee-regarding-the-outbreak-of-novel-coronavirus-(2019-ncov) (2020).

[3] Phelan AL, Katz R, Gostin LO (2020). The novel coronavirus originating in Wuhan, China: challenges for Global Health Governance. JAMA.

[4] Di Pierro F, Bertuccioli A, Cavecchia I (2020). Possible therapeutic role of a highly standardized mixture of active compounds derived from cultured Lentinula edodes mycelia (AHCC) in patients infected with 2019 novel coronavirus. Minerva Gastroenterol. Dietol..

[5] Zhou P (2020). A pneumonia outbreak associated with a new coronavirus of probable bat origin. Nature.

[6] Lu R (2020). Genomic characterisation and epidemiology of 2019 novel coronavirus: implications for virus origins and receptor binding. Lancet.

[7] Ranasinghe S (2016). Antiviral CD8(+) T cells restricted by human leukocyte antigen class II exist during natural HIV infection and exhibit clonal expansion. Immunity.

[8] Tang-Huau TL (2018). Human in vivo-generated monocyte-derived dendritic cells and macrophages cross-present antigens through a vacuolar pathway. Nat. Commun..

[9] Braciale TJ, Sun J, Kim TS (2012). Regulating the adaptive immune response to respiratory virus infection. Nat. Rev. Immunol..

[10] Bassing CH, Swat W, Alt FW (2002). The mechanism and regulation of chromosomal V(D)J recombination. Cell.

[11] Ni L (2020). Detection of SARS-CoV-2-specific humoral and cellular immunity in COVID-19 convalescent individuals. Immunity.

[12] Wen W (2020). Immune cell profiling of COVID-19 patients in the recovery stage by single-cell sequencing. Cell Discov..

[13] Weiskopf D (2020). Phenotype and kinetics of SARS-CoV-2-specific T cells in COVID-19 patients with acute respiratory distress syndrome. Sci. Immunol..

[14] Wu C (2020). Risk factors associated with acute respiratory distress syndrome and death in patients with coronavirus disease2019 pneumonia in Wuhan, China. JAMA Intern. Med..

[15] Braun J (2020). SARS-CoV-2-reactive T cells in healthy donors and patients with COVID-19. Nature.

[16] Grifoni A (2020). Targets of T cell responses to SARS-CoV-2 coronavirus in humans with COVID-19 disease and unexposed individuals. Cell.

[17] Mateus J (2020). Selective and cross-reactive SARS-CoV-2 T cell epitopes in unexposed humans. Science.

[18] Le Bert N (2020). SARS-CoV-2-specific T cell immunity in cases of COVID-19 and SARS, and uninfected controls. Nature.

[19] Huang IC (2011). Distinct patterns of IFITM-mediated restriction of filoviruses, SARS coronavirus, and influenza A virus. PLoS Pathog..

[20] Feeley EM (2011). IFITM3 inhibits influenza A virus infection by preventing cytosolic entry. PLoS Pathog..

[21] Pfaender S (2020). LY6E impairs coronavirus fusion and confers immune control of viral disease. Nat. Microbiol..

[22] Ashburner M (2000). Gene ontology: tool for the unification of biology. The Gene Ontology Consortium. Nat. Genet.

[23] Gene Ontology, C. The Gene Ontology resource: enriching a GOld mine. *Nucleic Acids Res.* **49**, D325–D334 (2020). 10.1093/nar/gkaa1113 PMC7779012 33290552

[24] van der Windt GJ (2012). Mitochondrial respiratory capacity is a critical regulator of CD8+ T cell memory development. Immunity.

[25] Geltink RIK, Kyle RL, Pearce EL (2018). Unraveling the complex interplay between T cell metabolism and function. Annu Rev. Immunol..

[26] Henning AN, Roychoudhuri R, Restifo NP (2018). Epigenetic control of CD8(+) T cell differentiation. Nat. Rev. Immunol..

[27] Chen S, Hu Z, He H, Liu X (2018). Fatty acid elongase7 is regulated via SP1 and is involved in lipid accumulation in bovine mammary epithelial cells. J. Cell Physiol..

[28] Haas MJ, Horani MH, Wong NC, Mooradian AD (2004). Induction of the apolipoprotein AI promoter by Sp1 is repressed by saturated fatty acids. Metabolism.

[29] Li Y (2019). Sp1 is involved in vertebrate LC-PUFA biosynthesis by upregulating the expression of liver desaturase and elongase genes. Int. J. Mol. Sci..

[30] Zhou R (2020). Acute SARS-CoV-2 infection impairs dendritic cell and T cell responses. Immunity.

[31] Kondo Y (2009). Hepatitis C virus infection of T cells inhibits proliferation and enhances fas-mediated apoptosis by down-regulating the expression of CD44 splicing variant 6. J. Infect. Dis..

[32] Chigbu DI, Loonawat R, Sehgal M, Patel D, Jain P (2019). Hepatitis C virus infection: host(-)virus interaction and mechanisms of viral persistence. Cells.

[33] Joeckel LT (2011). Mouse granzyme K has pro-inflammatory potential. Cell Death Differ..

[34] Afonina IS (2011). Granzyme B-dependent proteolysis acts as a switch to enhance the proinflammatory activity of IL-1alpha. Mol. Cell.

[35] Cooper DM, Pechkovsky DV, Hackett TL, Knight DA, Granville DJ (2011). Granzyme K activates protease-activated receptor-1. PLoS One.

[36] Voskoboinik I, Whisstock JC, Trapani JA (2015). Perforin and granzymes: function, dysfunction and human pathology. Nat. Rev. Immunol..

[37] Krensky AM, Clayberger C (2009). Biology and clinical relevance of granulysin. Tissue Antigens.

[38] Pardo J, Balkow S, Anel A, Simon MM (2002). The differential contribution of granzyme A and granzyme B in cytotoxic T lymphocyte-mediated apoptosis is determined by the quality of target cells. Eur. J. Immunol..

[39] Ball TB (2007). Polymorphisms in IRF-1 associated with resistance to HIV-1 infection in highly exposed uninfected Kenyan sex workers. AIDS.

[40] Sivro A, Su RC, Plummer FA, Ball TB (2013). HIV and interferon regulatory factor 1: a story of manipulation and control. AIDS Res Hum. Retroviruses.

[41] Rao RR, Li Q, Odunsi K, Shrikant PA (2010). The mTOR kinase determines effector versus memory CD8+ T cell fate by regulating the expression of transcription factors T-bet and Eomesodermin. Immunity.

[42] Araki K (2009). mTOR regulates memory CD8 T-cell differentiation. Nature.

[43] Stuart T (2019). Comprehensive integration of single-cell data. Cell.

[44] Ren X (2021). Insights gained from single-cell analysis of immune cells in the tumor microenvironment. Annu Rev. Immunol..

[45] Sandu I (2020). Landscape of exhausted virus-specific CD8 T cells in chronic LCMV infection. Cell Rep..

[46] Yu G, Wang LG, Han Y, He QY (2012). clusterProfiler: an R package for comparing biological themes among gene clusters. OMICS.

[47] Carlson, M. org.Hs.eg.db: Genome wide annotation for Human. R package version 3.8.2. (2019).

[48] Ma A (2020). IRIS3: integrated cell-type-specific regulon inference server from single-cell RNA-Seq. Nucleic Acids Res..

[49] Organization, W. H. *WHO COVID-19 Therapeutic Trial Synopsis*, https://www.who.int/blueprint/priority-diseases/key-action/COVID-19_Treatment_Trial_Design_Master_Protocol_synopsis_Final_18022020.pdf (2020).

