# Supplementary material for: Dynamics of TCR repertoire and T cell function in COVID-19 convalescent individuals
Source: Cell Discov. 2021 Sep 28;7:89. doi: 10.1038/s41421-021-00321-x (PMC8476510; doi:10.1038/s41421-021-00321-x)
Supplement: Supplementary file 1 — Supplementary Information [file 41421_2021_321_MOESM1_ESM.pdf]

Supplemental information

Supplementary Fig. S1 Clonal diversity of samples

The clone diversity was displayed via the TCR diversity, percentage of each sample's clone diversity = (TCR classification count / total cell number) * 100%. Doughnuts (**a**) and column (**b**) showed the T-cell expanded clone and clonal diversity percentage of the representative sample from each group. The colored wedge represents the percentage of not expanded (white/grey), clonal expanded 2 to 4 times (orange), 5 to 9 times (blue), 10 to 49 times (green), 50 to 99 (purple), or more than 100 times (red) in total TCRs in the pool. DC, Discharged group; EFU, Early Follow-up group; LFU, Late Follow-up group; HD, Healthy Donor group.

Supplementary Fig. S2 V-J gene pairing

Alpha chains V-J pairing of TCRs from representative samples of the HD group (HD#3) (**a**), the Discharged group (Discharged#2) (**b**) and the Follow-up group (Follow-up#3) (**c**). Beta chains V-J pairing of TCRs from representative samples of the HD group (HD#3) (**upper panel**), the Discharged group (Discharged#2) (**middle panel**) and the Follow-up group (Follow-up#3) (**down panel**).

Supplementary Fig. S3 Batch effect correction of scRNA-seq data

The merged scRNA-seq data of samples without batch effect **(a)** and with batch effect via “FindIntergrationAnchors” and “IntegrateData” functions of Seurat package (V3.6.0) (**b**) (n = 6 in Discharged and HD groups, n = 7 in Follow-up group). DC, Discharged group; FU, Follow-up group; HD, Healthy Donor group.

Supplementary Fig. S4 *CD3E*, *CD4* and *CD8A* expression in total TCR paired scRNA-seq data

The expression of *CD3E*, *CD4* and *CD8A* in total TCR paired scRNA-seq integrated data was displayed by violin plot **(a)** and feature plot (**b**) (n = 6 in Discharged and HD groups, n = 7 in Follow-up group). Teff, effector T; Tex, exhausted T, Tcm, central memory T; Tem, effector memory T; Tm, memory T, Tn, naïve T; Treg, regulatory T.

Supplementary Fig. S5 Top 20 clones’ projection in total TCR paired scRNA-seq data

UMAP showing the top 20 clones’ distribution in clusters (**left panel**) and clusters’ distribution (**right panel**). Teff, effector T; Tex, exhausted T, Tcm, central memory T; Tem, effector memory T; Tm, memory T, Tn, naïve T; Treg, regulatory T.

Supplementary Fig. S6 Gene expression in each cluster of total TCR paired scRNA-seq data

Bubble plot showing the expression level of marker genes in the clusters. The clusters were identified with expressed genes: 0: $CD4^{+}CCR7^{+}LEF1^{hi}$ naïve T ($SELL^{+}CCR7^{+}LEF1^{hi}TCF^{+}$); 1: $CD4^{+}CCR7^{+}$ naïve T ($SELL^{+}CCR7^{+}LEF1^{+}TCF^{+}$); 2: $CD8A^{+}GZMH^{hi}$ cytotoxic effector T ($SELL^{-}CCR7^{-}GZMA^{+}GZMB^{hi}PRF1^{hi}GZMH^{hi}$); 3: $CD8A^{+}$ exhausted T ($SELL^{-}CCR7^{-}GZMA^{+}GZMK^{+}$ $GZMB^{-}$); 4: $CD8A^{+}JUN^{+}LEF1^{+}$ naïve T ($SELL^{+}CCR7^{hi}LEF1^{+}TCF7^{+}JUN^{+}$); 5: $CD8A^{+}GZMK^{+}$ effector memory T ($SELL^{-}CCR7^{-}GZMK^{+}$); 6: $CD8A^{+}GNLY^{hi}$ cytotoxic effector T ($SELL^{-}CCR7^{-}GZMA^{+}GZMB^{hi}PRF1^{hi}GZMH^{+}GNLY^{hi}$); 7: $CD4^{+}IL7R^{hi}$ effector T ($SELL^{-}CCR7^{-}IL7R^{hi}$); 8: $CD4^{+}ANXA1^{+}$ effector T ($SELL^{-}CCR7^{-}JUNB^{+}EMP3^{+}ANXA1^{+}$); 9: $CD4^{+}$ Treg ($FOXP3^{hi}IL10RA^{hi}$); 10: $CD8A^{+}KLRB1^{hi}CXCR4^{hi}$ effector-exhausted intermediated T ($GZMA^{lo}GZMB^{-}GZMK^{+}PRF1^{lo}KLRB1^{hi}CXCR4^{hi}$); 11: $CD4^{+}CDC25B^{hi}JUNB^{+}$ memory T ($SELL^{hi}CCR7^{lo}CDC25B^{hi}JUNB^{+}$); 12: $CD4^{+}GZMH^{+}$ effector ($SELL^{-}CCR7^{-}GZMH^{+}$); 13: $CD4^{+}S100A8^{hi}S100A9^{hi}$ Th22 ($AHR^{+}S100A8^{hi}S100A9^{hi}$); 14: $CD8A^{+}LEF1^{lo}$ naïve T ($SELL^{lo}CCR7^{lo}LEF1^{lo}TCF7^{lo}$); 15: $CD8A^{+}$ proliferative T subset ($CSK2^{+}LIG1^{+}MKI67^{+}MCM2^{+}MCM3^{+}STMN1^{hi}$ $PDCD1^{lo}TOX^{lo}HAVCR2^{lo}LAG3^{lo}$); 16: $CD8A^{+}CXCR5^{+}$ central memory T ($SELL^{lo}CCR7^{lo}CXCR5^{+}$).

Supplementary Fig. S7 *CD3E*, *CD4* and *CD8A* expression in the top 20 TCRs paired scRNA-seq data

The expression of *CD3E*, *CD4* and *CD8A* in the top 20 TCR clonotypes paired scRNA-seq integrated data was displayed by violin plot (**a**) and feature plot (**b**) (n = 6 in Discharged and HD groups, n = 7 in Follow-up group). Teff, effector T; Tex, exhausted T, Tcm, central memory T; Tem, effector memory T; Tm, memory T, Tn, naïve T; Treg, regulatory T.

Supplementary Fig. S8 Samples distribution in the top 20 TCRs paired scRNA-seq data

(**a**) UMAP showing the samples' distribution in clusters. (**b**) Bar plot showing the compositions of each cluster in three groups. Teff, effector T; Tex, exhausted T, Tcm, central memory T; Tem, effector memory T; Tm, memory T, Tn, naïve T; Treg, regulatory T; DC, Discharged group; FU, Follow-up group; HD, Healthy Donor group.

Supplementary Fig. S9 Gene expression in each cluster of the top 20 TCRs paired scRNA-seq data

Bubble plot showing the expression level of marker genes in the clusters. The clusters were annotated with the expressed genes: 0: $CD8A^{+}$ cytotoxic effector T ($GZMB^{+}PRF1^{+}$); 1: $CD8A^{+}GZMB^{lo}PRF1^{lo}$ cytotoxic effector T ($SELL^{-}CCR7^{-}GZMB^{lo}PRF1^{lo}$); 2: $CD8A^{+}GZMB^{hi}PRF1^{hi}$ cytotoxic effector T ($GZMB^{hi}PRF1^{hi}$); 3: $CD8A^{+}GZMA^{int}GZMB^{hi}PRF1^{hi}$ cytotoxic effector T ($SELL^{-}CCR7^{-}GZMA^{int}GZMB^{hi}PRF1^{hi}$); 4: $CD4^{+}KLRB1^{+}GZMH^{+}$ effector T ($SELL^{-}CCR7^{-}$

$KLRB1^{+}GZMH^{+}$); 5: $CD8A^{+}GZMA^{lo}GZMK^{lo}$ effector T ($SELL^{-}CCR7^{-}GZMA^{lo}GZMK^{lo}$); 6: $CD8A^{+}GZMK^{+}$ effector memory T ($SELL^{lo}CCR7^{lo}GZMK^{+}$); 7: $CD8A^{+}KLRB1^{hi}$ $CXCR4^{hi}$ terminal differentiation T ($ID2^{hi}PRDM1^{+}IL7R^{hi}KLRB1^{hi}CXCR4^{hi}$); 8: $CD8A^{+}CD7^{+}GZMA^{lo}GZMB^{hi}PRF1^{hi}$ effector-exhaustion intermediate T ($HAVCR2^{+}LAG3^{lo}CD7^{+}GZMA^{lo}GZMB^{hi}PRF1^{hi}$); 9: degranulation T ($JUND^{hi}S100A8^{hi}S100A9^{hi}$); 10: $CD8A^{+}CXCR5^{+}$ central memory T ($SELL^{+}CCR7^{-}JUN^{+}CXCR5^{+}$); 11: $CD8A^{+}GZMB^{int}PRF1^{int}$ cytotoxic effector T ($SELL^{lo}CCR7^{-}GZMB^{int}PRF1^{int}$); 12: $CD8A^{+}CD160^{hi}$ effector T ($SELL^{-}CCR7^{-}$ $CD160^{hi}$); 13: $CD8A^{+}$ high cytotoxic potential effector T ($SELL^{-}CCR7^{-}PRF1^{+}ITGAM^{+}$); 14: $CD8A^{+}$ terminal proliferative T ($CSK2^{+}LIG1^{+}MKI67^{+}MCM2^{+}MCM3^{+}STMN1^{hi}$); 15: $CD4^{+}$ exhausted T ($SELL^{-}CCR7^{-}PDCD1^{+}TOX^{+}PRDM1^{+}GZMA^{hi}GZMK^{hi}$); 16: $CD4^{+}IFITM2^{hi}$ effector T ($SELL^{-}CCR7^{-}IFITM1^{+}IFITM2^{hi}$).

Supplementary Fig. S10 Gene concept network analysis of differentially expressed genes of $CD3E^{+}CD8A^{+}CD4^{-}$ clusters in top 20 TCRs paired scRNA-seq data from Discharged group *vs*. HD group group

The bubble plot of selected granzyme- and interferon – related genes' expression level in of $CD3E^{+}CD8A^{+}CD4^{-}$ clusters in top 20 clonotypes from Discharged group *vs*. HD group (n = 6 per group).

Supplementary Fig. S11 Biological process and GSEA analysis for total *CD3E*$^{+}$*CD8A*$^{+}$*CD4*$^{-}$ clusters from Discharged group *vs*. HD group

(**a**) Using GSEA to analyze expressed genes from *CD3E*$^{+}$*CD8A*$^{+}$*CD4*$^{-}$ clusters, 2 gene sets of interferon response up-regulated in Discharged group comparing with HD group (n = 6 per group). NES, normalized ES; FDR, false discovery rate; NOM p, normalized p value. (**b**) Top 20 BP enrichment analysis of DEGs from *CD3E*$^{+}$*CD8A*$^{+}$*CD4*$^{-}$ clusters, which up-regulated in Discharged group from Discharged *vs*. HD integrated data (n = 6 per group).

Supplementary Fig. S12 GSEA analysis for total *CD3E*$^{+}$*CD8A*$^{+}$*CD4*$^{-}$ clusters from Follow-up group *vs*. HD group

Using GSEA to analyze expressed genes from *CD3E*$^{+}$*CD8A*$^{+}$*CD4*$^{-}$ clusters, 3 gene sets about metabolism up-regulated in total Follow-up group from total Follow-up *vs.* HD (n = 6 in HD group, n = 7 in Follow-up group). NES, normalized ES; FDR, false discovery rate; NOM p, normalized p value.

Supplementary Fig. S13 GSEA analysis for top 20 TCRs *CD3E*$^{+}$*CD8A*$^{+}$*CD4*$^{-}$ clusters from Early Follow-up group *vs*. HD group

Top 20 BP enrichment analysis of DEGs from *CD3E*$^{+}$*CD8A*$^{+}$*CD4*$^{-}$ clusters, which up regulated in Early Follow-up group from Early Follow-up *vs*. HD integrated data (n = 3 in Early Follow-up groups, n = 6 in HD group).

Supplementary Fig. S14 Transcription factor analysis of *CD8A*$^{+}$*GZKM*$^{+}$ effector memory T cells from Late Follow-up group via IRIS3 from IRIS3 website

Supplementary Table S1. Clinical and Pathological Characteristics of the COVID-19 Patients and Healthy Donors

Supplementary Table S2. The scRNA-seq / scTCR-seq protocol and data characteristics in the current study via Cell Ranger analysis

Supplementary Table S3. The scRNA-seq / scTCR-seq protocol and data characteristics in the current study via Seurat package

Supplementary Table S4. Performed scRNA-seq and paired TCR cell count in each group

Supplementary Table S5. The unique-clone and multi-clone percentage in each group

Supplementary Fig. S1

a

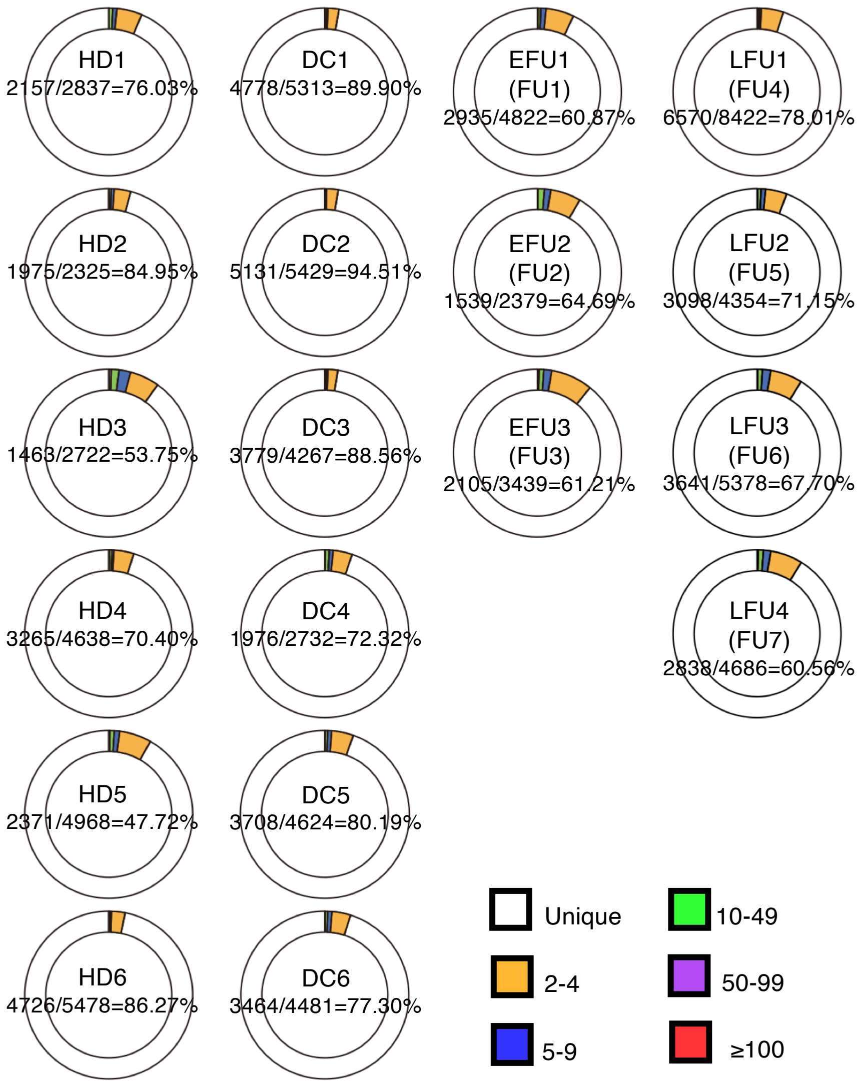

b

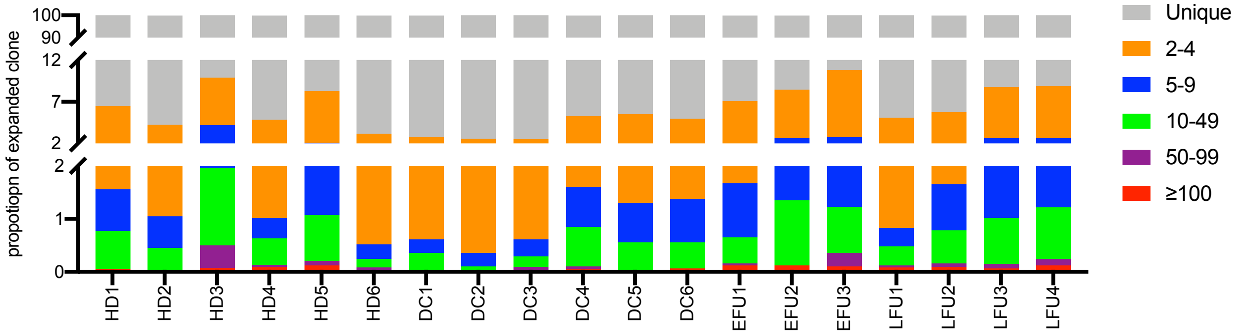

Supplementary Fig. S2

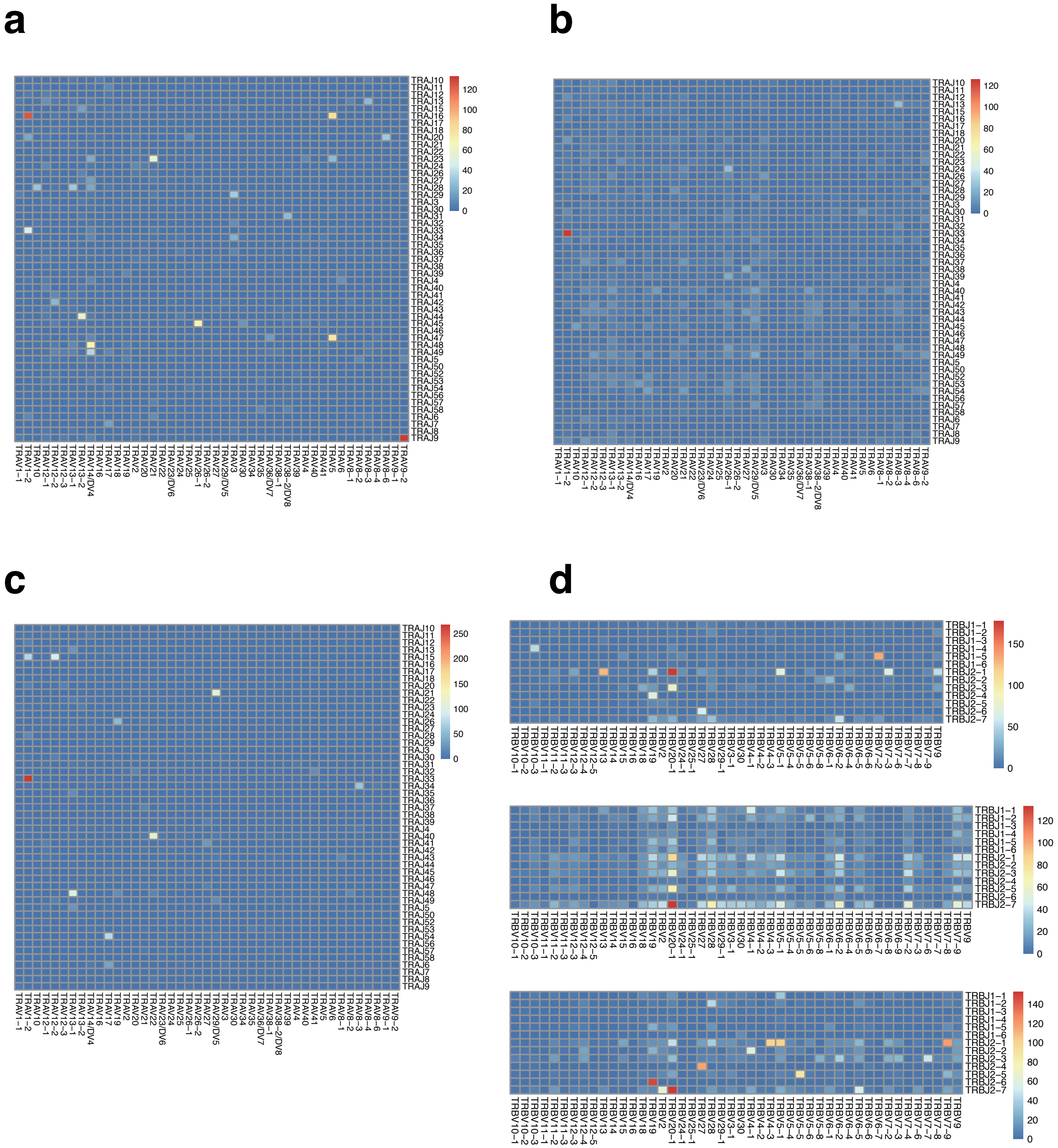

Supplementary Fig. S3

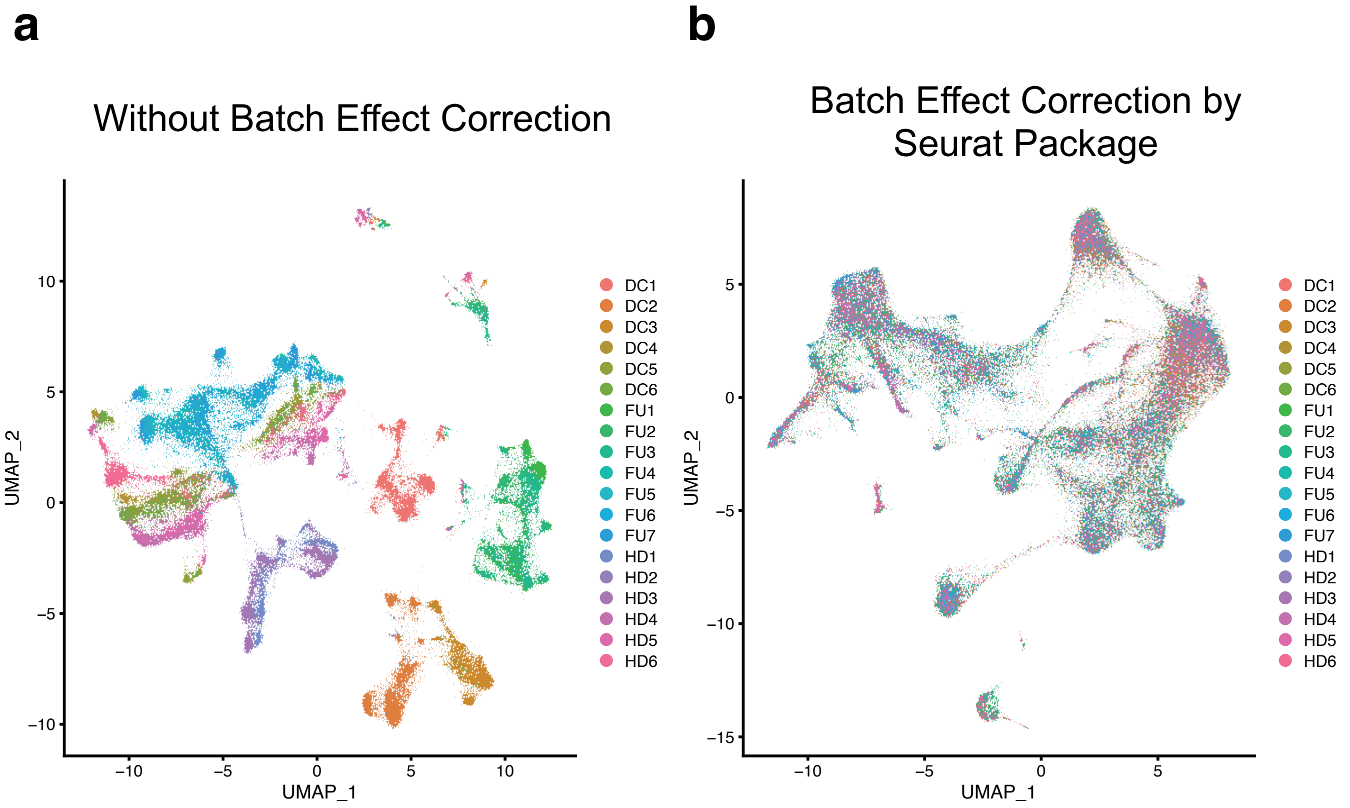

Supplementary Fig. S4

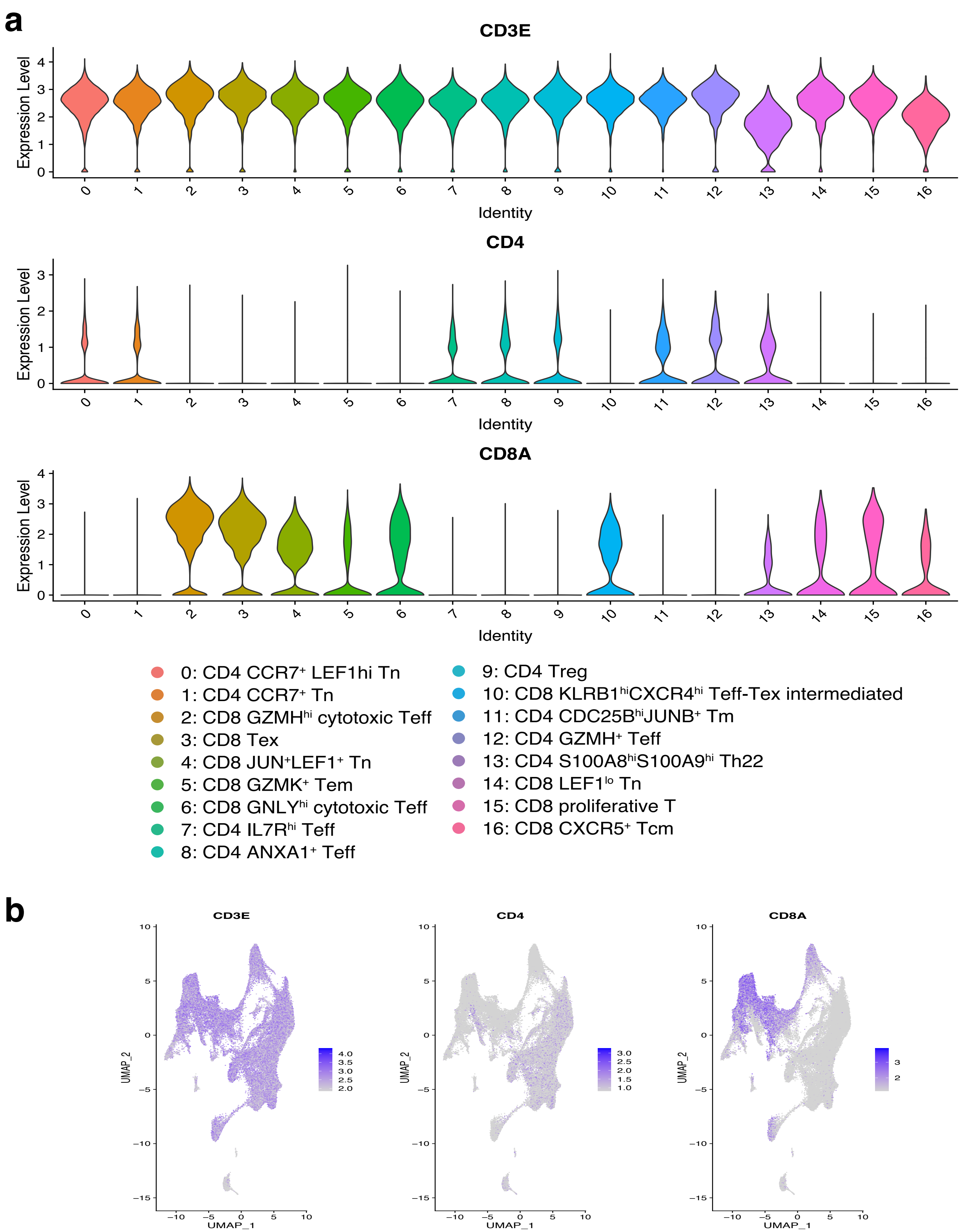

Supplementary Fig. S5

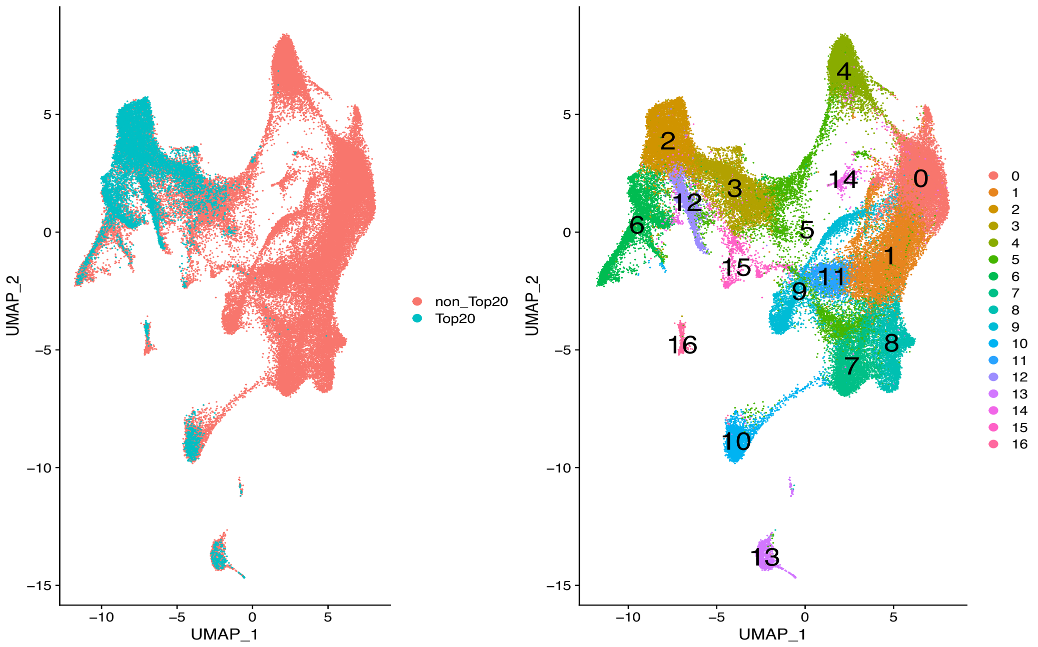

- 0: CD4 CCR7^{+} LEF1hi Tn
- 1: CD4 CCR7^{+} Tn
- 2: CD8 GZMH^{hi} cytotoxic Teff
- 3: CD8 Tex
- 4: CD8 JUN^{+}LEF1^{+} Tn
- 5: CD8 GZMK^{+} Tem
- 6: CD8 GNLY^{hi} cytotoxic Teff
- 7: CD4 IL7R^{hi} Teff
- 8: CD4 ANXA1^{+} Teff
- 9: CD4 Treg
- 10: CD8 KLRB1^{hi}CXCR4^{hi} Teff-Tex intermediated
- 11: CD4 CDC25B^{hi}JUNB^{+} Tm
- 12: CD4 GZMH^{+} Teff
- 13: CD4 S100A8^{hi}S100A9^{hi} Th22
- 14: CD8 LEF1^{lo} Tn
- 15: CD8 proliferative T
- 16: CD8 CXCR5^{+} Tcm

Supplementary Fig. S6

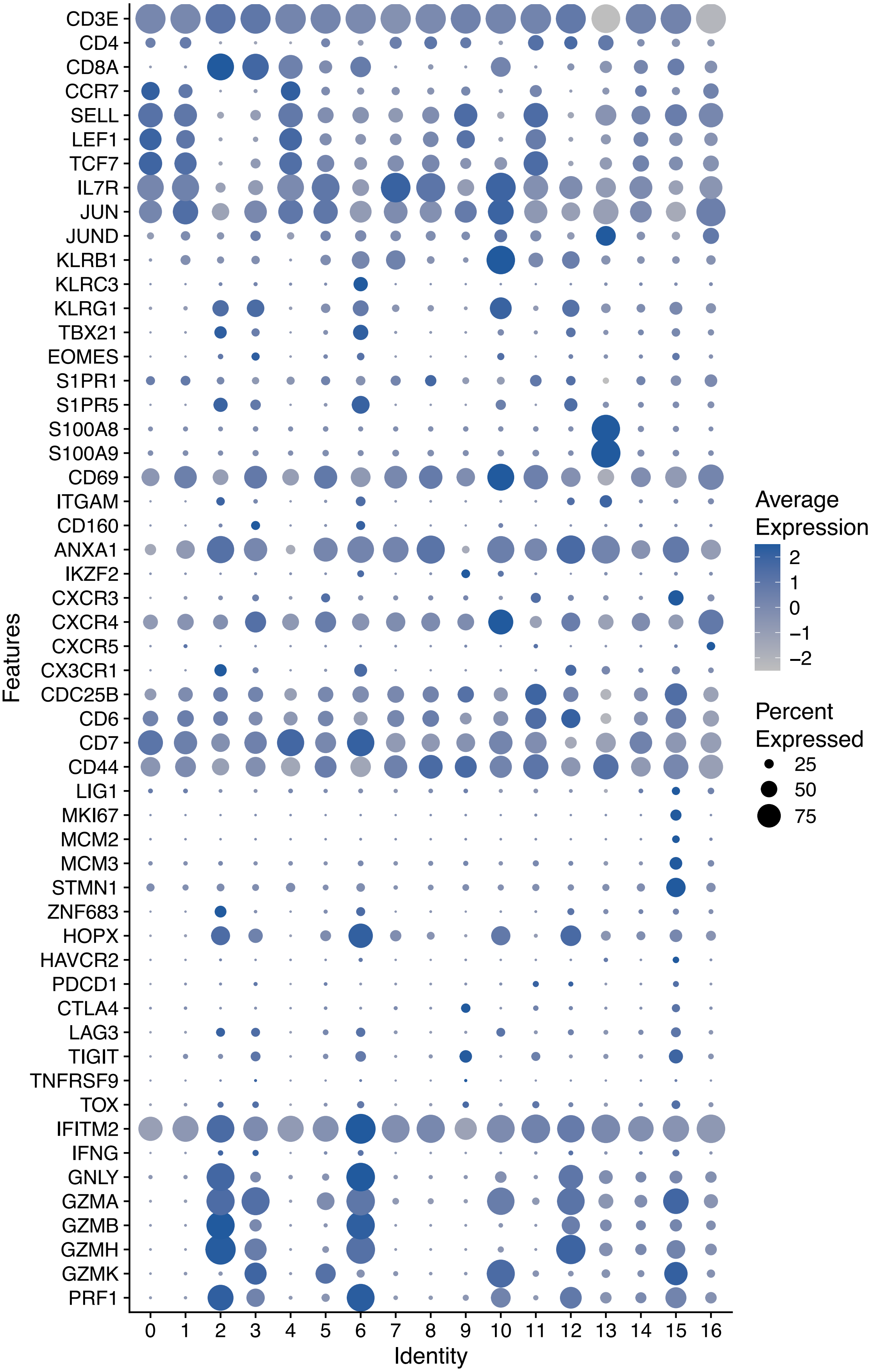

Supplementary Fig. S7

a

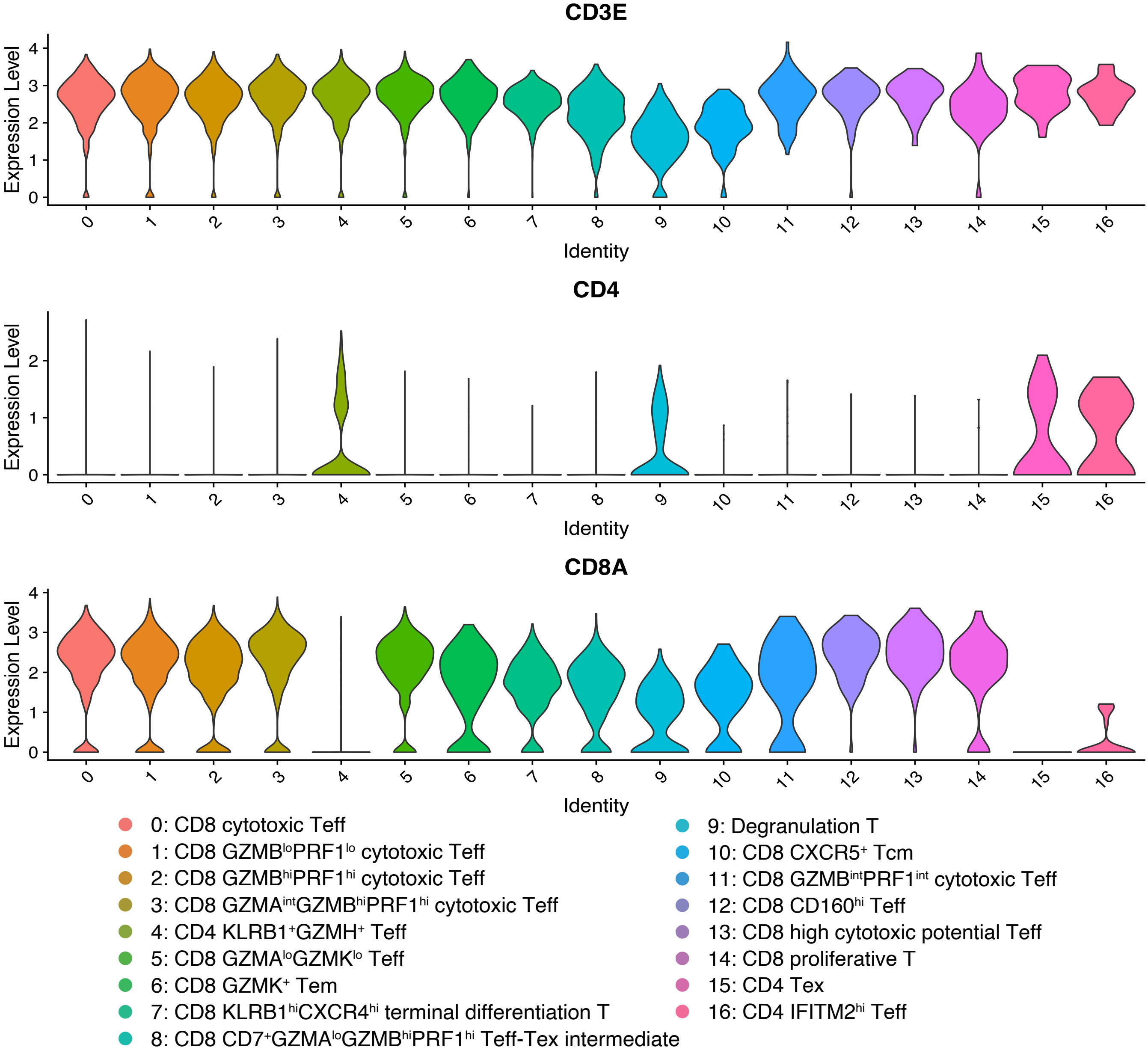

b

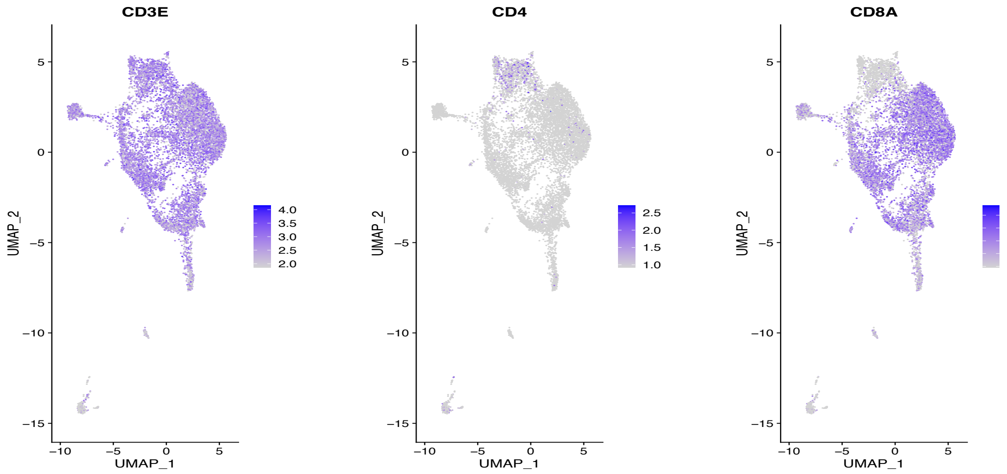

Supplementary Fig. S8

a

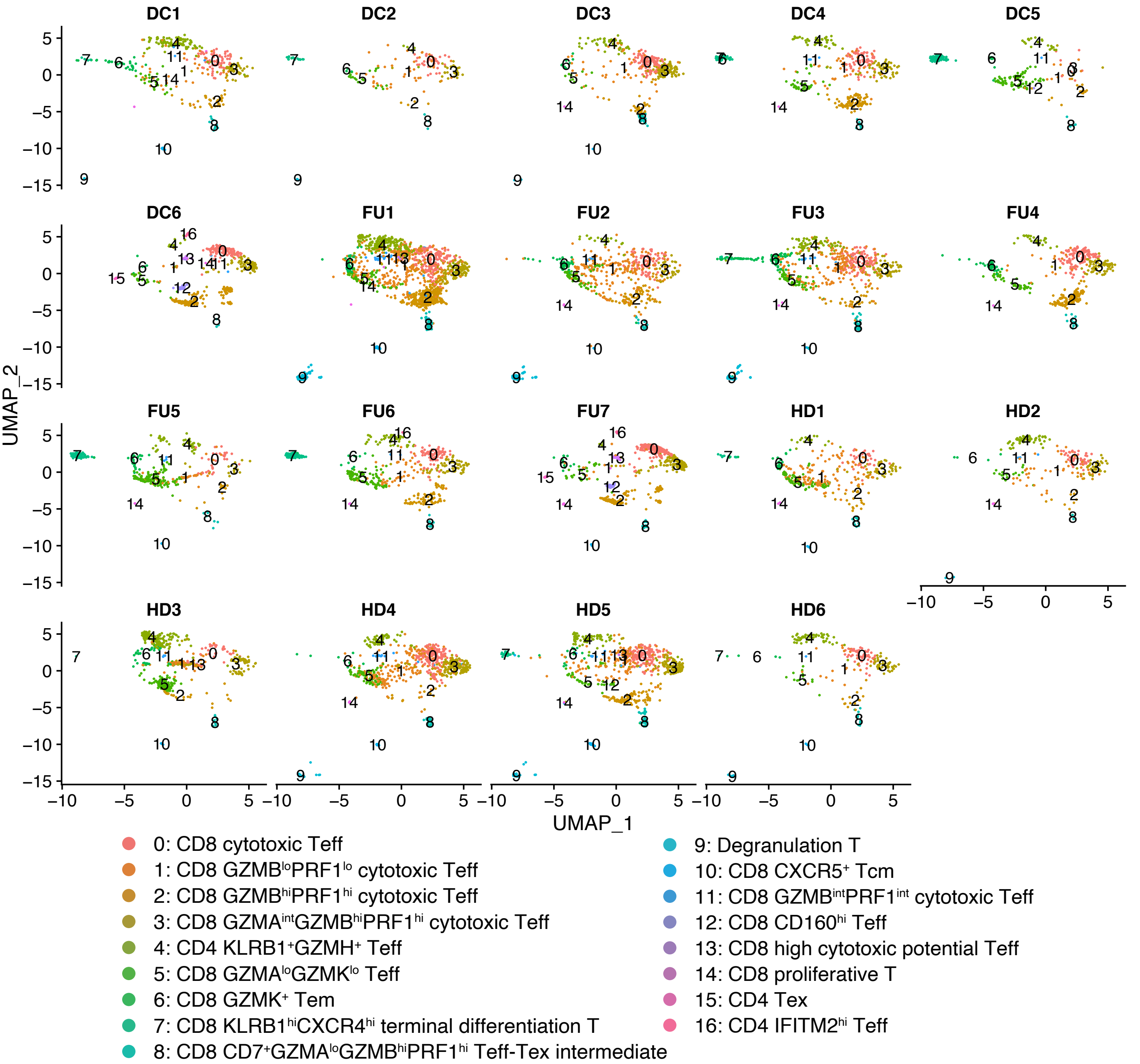

b

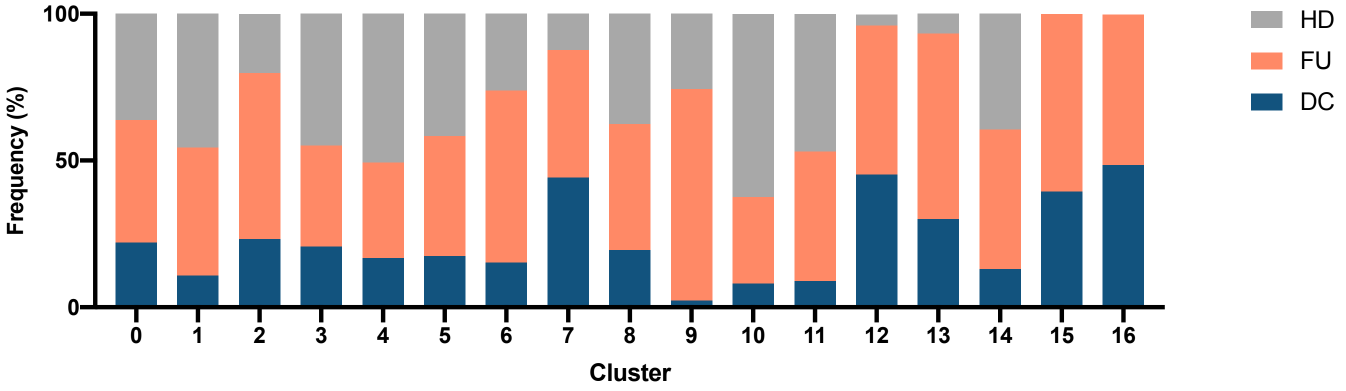

Supplementary Fig. S9

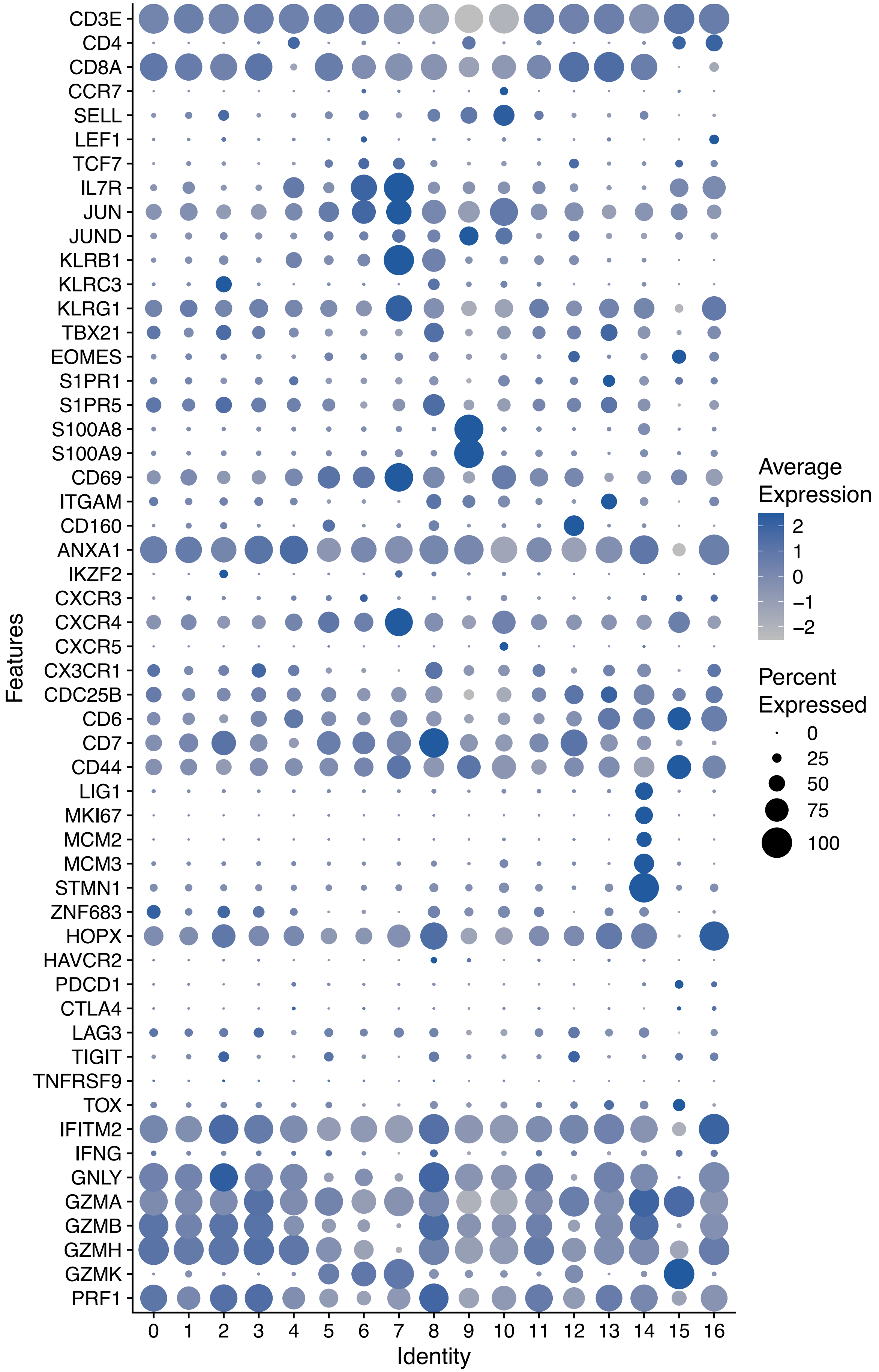

Supplementary Fig. S10

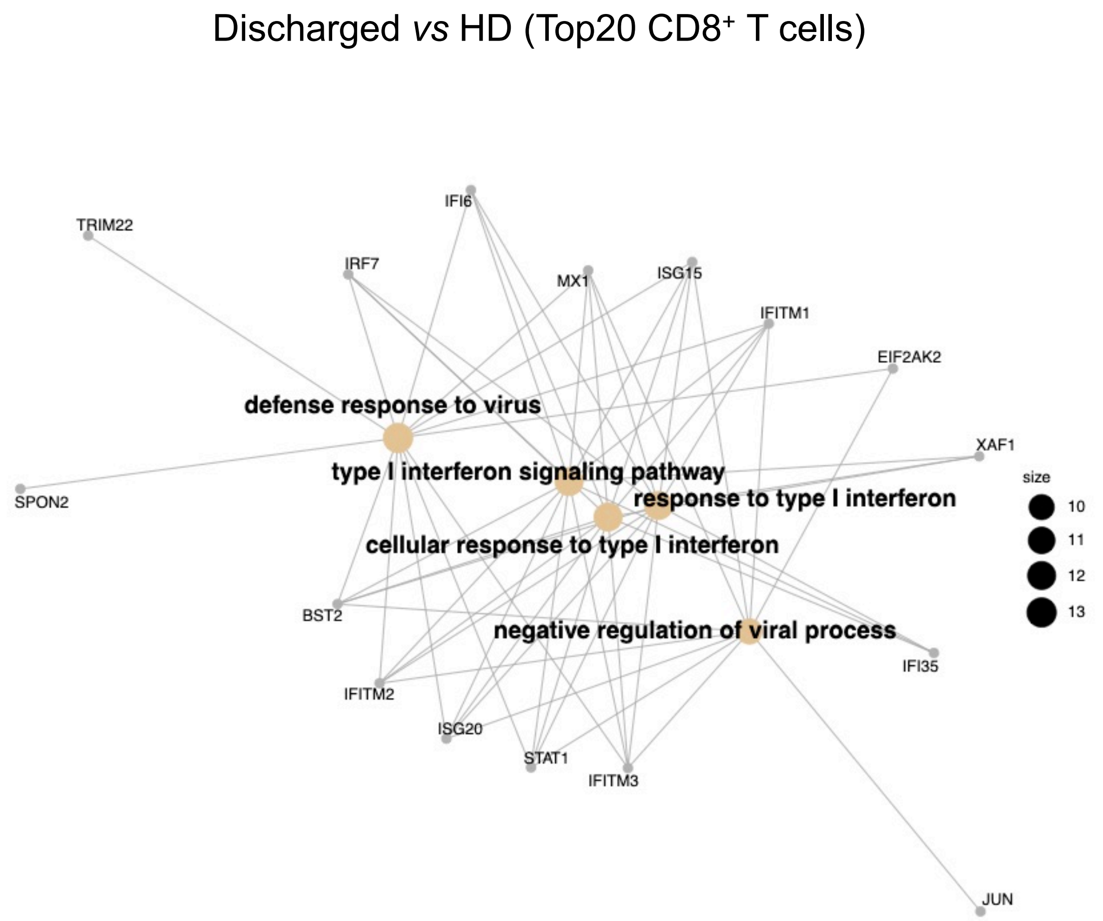

Supplementary Fig. S11

a

Discharged *vs* HD (Total CD8$^+$ T cells)

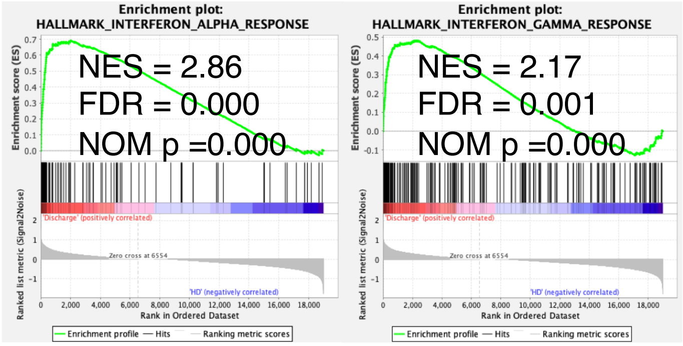

b

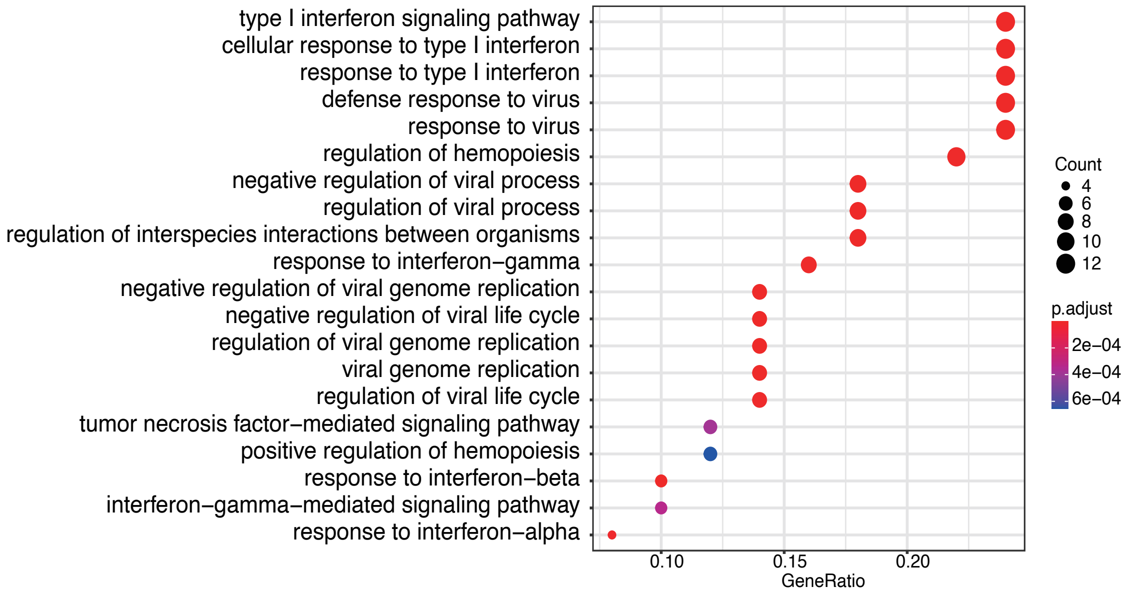

Supplementary Fig. S12

Follow-up *vs* HD (Total CD8$^+$ T cells)

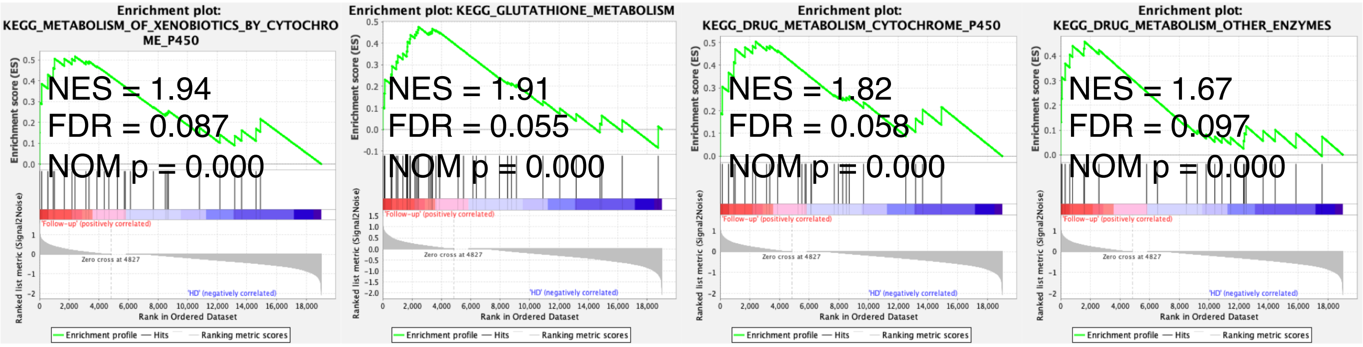

Supplementary Fig. S13

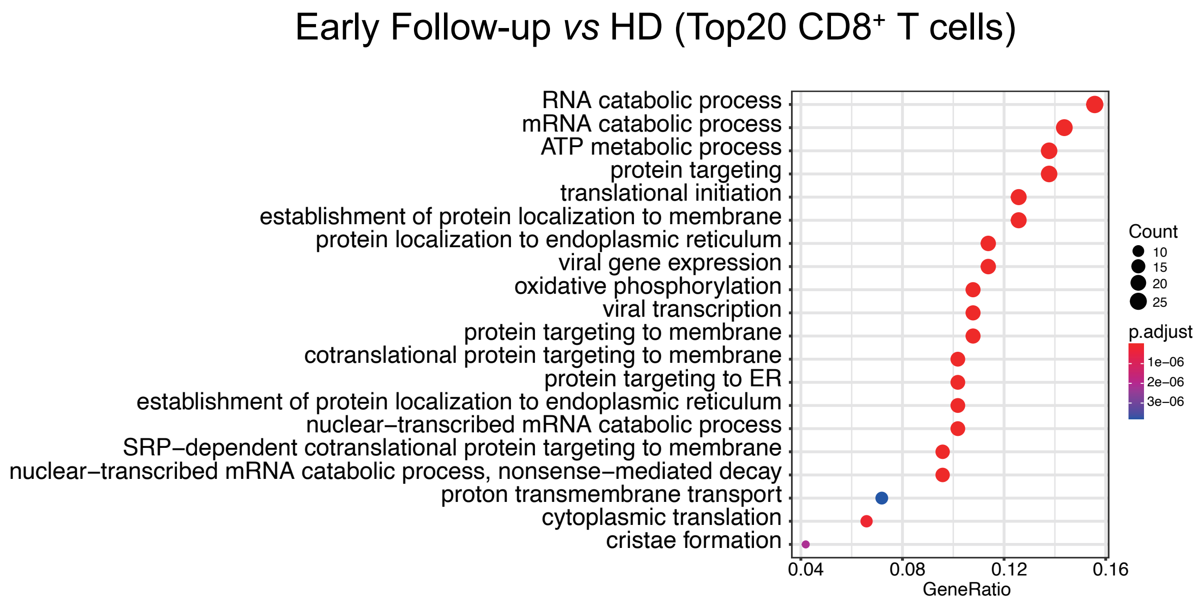

Supplementary Fig. S14

Regulon Specificity Score Scatter Plot for *CD8A^{+}GZMK^{+}* Tem of Late Follow-up

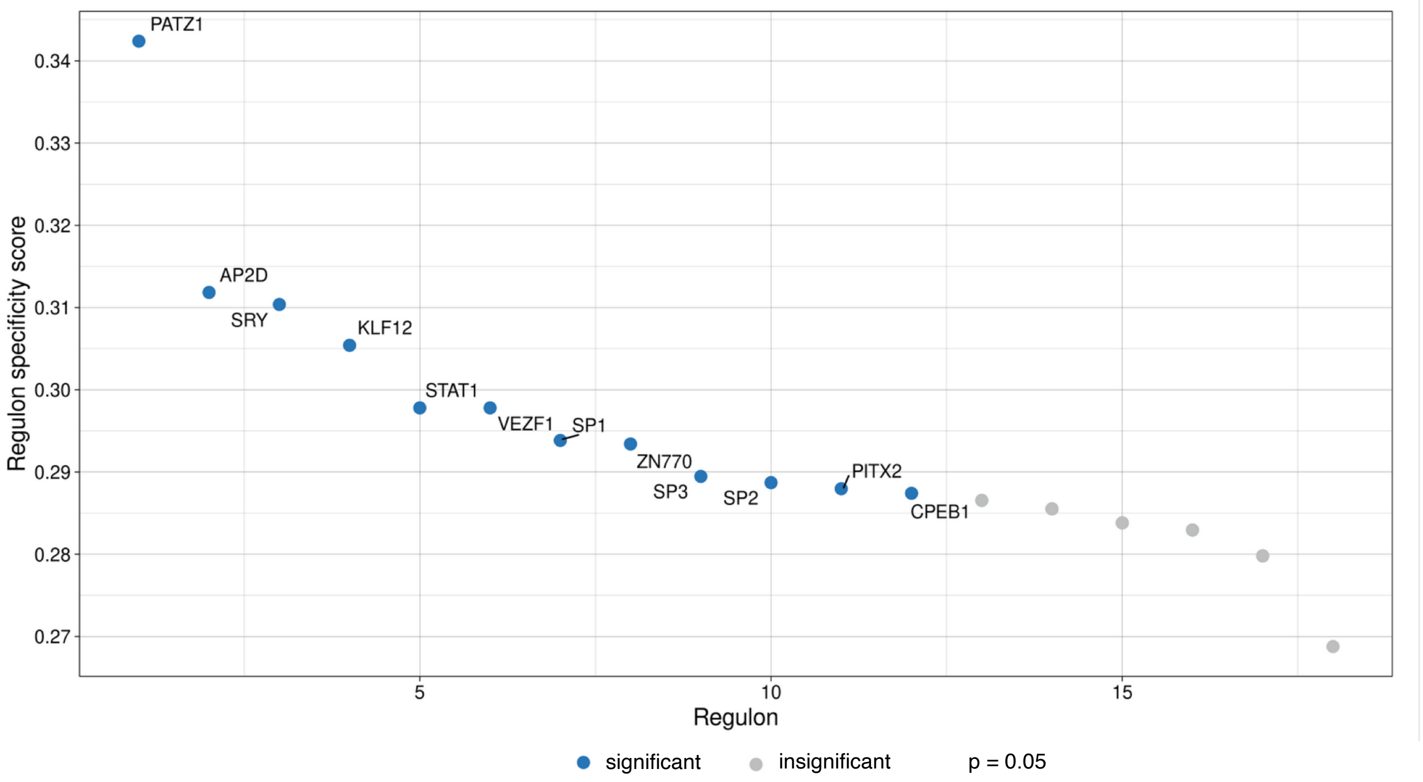

Supplementary Table S1 Clinical and Pathological Characteristics of the COVID-19 Patients and Healthy Donors

	HD1	HD2	HD3	HD4	HD5	HD6	DC1	DC2	DC3	DC4	DC5	DC6	EFU1 (FU1)	EFU2 (FU2)	EFU3 (FU3)	LFU1 (FU4)	LFU2 (FU5 =DC4)	LFU3 (FU6 =DC5)	LFU4 (FU7 =DC6)
**Sex**	F	M	M	F	F	M	F	M	M	M	F	F	M	F	M	M	M	F	F
**Age**	37	27	27	66	36	31	29	35	32	42	49	32	51	44	46	35	42	49	32
**Days in Hospital**	-	-	-	-	-	-	27	20	14	14	17	18	16	15	19	20	14	17	18
**Sampling Time**	-	-	-	-	-	-	-6	0	0	1	-2	0	7	7	7	30	40	19	30
**S Antigen-IgG**	-	-	-	-	-	-	P(+)	P(+)	P(+)	-	-	-	P(+++)	P(+++)	P(+++)	-	-	-	-
**S Antigen-IgM**	-	-	-	-	-	-	P(+)	P(+)	N	-	-	-	P(+)	P(+)	P(+)	-	-	-	-
**BT NA Test**	-	-	-	-	-	-	P	P	P	P	P	P	P	P	P	P	P	P	P
**Outcome of Disease**	-	-	-	-	-	-	CR	CR	CR	CR	CR	CR	CR	CR	CR	CR	CR	CR	CR
**Discharge NA Test**	N	N	N	N	N	N	N	N	N	N	N	N	N	N	N	N	N	N	N
**Disease Severity** [27]	0	0	0	0	0	0	3	3	3	3	3	3	3	3	3	3	3	3	3
**HLA-A Antigen**	-	-	-	-	-	-	HLA-A*02:07, HLA-A*26:01	-	-	HLA-A*02:06, HLA-A*02:06	HLA-A*11:01, HLA-A*11:01	HLA-A*11:01, HLA-A*11:01	HLA-A*02:01, HLA-A*24:02	HLA-A*02:03, HLA-A*24:02	HLA-A*11:01, HLA-A*11:01	HLA-A*02:06, HLA-A*02:07	HLA-A*02:06, HLA-A*02:06	HLA-A*11:01, HLA-A*11:01	HLA-A*11:01, HLA-A*11:01

HD, Healthy donor group; DC, Discharged group; EFU, Early Follow-up group; LFU, Late Follow-up; F, female; M, male; S antigen, Novel Coronavirus spike antigen; P, positive; N, negative; BT, before treatment; NA, nucleic acid; -, not performed; CR, complete recovery.

Supplementary Table S2 The scRNA-seq / scTCR-seq protocol and data characteristics in the current study via Cell Ranger analysis

Donor	Protocol	Number of Reads	Estimated Cell	Median Gene/Cell	Mean Reads/Cell	Median UMI/Cell	Fraction Reads in Cells(%)	Estimated T Cell	Cells with Productive V-J pair	Mean Read Pairs/Cell	Paired Clonotype Diversity
HD1	5'+VDJ	311,311,571	11,067	1,082	28,130	3,086	85.5%	4,924	2,865	6,018	140.29
HD2	5'+VDJ	331,176,719	7,099	1,227	46,651	3,855	90.1%	3,704	2,351	8,836	685.84
HD3	5'+VDJ	276,137,518	8,228	1,320	33.561	3,983	91.4%	4,128	2,746	10,548	121.07
HD4	5'+VDJ	849,896,247	10,016	1,752	84,854	5,092	97.5%	5,475	4,502	6,381	68.44
HD5	5'+VDJ	859,323,606	11,556	1,699	74,362	4,676	97.0&	5,876	4,750	6,448	38.32
HD6	5'+VDJ	823,645,256	11,607	1,643	70,961	4,914	97.8%	6,089	5,403	5,580	994.46
DC1	5'+VDJ	294,905,555	12,058	1,056	24.457	3,030	85.6%	6,715	5,361	4,240	1,914.36
DC2	5'+VDJ	278,083,003	13,965	1,136	19,913	2,589	92.8%	7,113	5,452	4,016	3,871.36
DC3	5'+VDJ	335,845,056	9,764	994	34,396	2,514	95.1%	6,914	4,299	4,006	820.78
DC4	5'+VDJ	833,182,912	9,552	1,418	87,226	4,685	94,6%	3,848	2,646	9,629	225.69
DC5	5'+VDJ	789,803,278	12,264	1,499	64,400	5,224	94,8%	6,349	4,457	5,289	700.77
DC6	5'+VDJ	857,408,210	10,041	1,634	85,391	5,065	97.1%	5,771	4,411	5,772	265,24
EFU1 (FU1)	5'+VDJ	NA	NA	NA	NA	NA	NA	5,737	4,857	5,249	70.84
EFU2 (FU2)	5'+VDJ	NA	NA	NA	NA	NA	NA	2,854	2,392	8,498	145.6
EFU3 (FU4)	5'+VDJ	NA	NA	NA	NA	NA	NA	5,004	3,463	4,966	155.24
LFU1 (FU4)	5'+VDJ	985,510,175	20,195	1,422	48,800	4,709	95.9%	10,154	8,088	3,125	431,68
LFU2 (FU5=DC4)	5'+VDJ	904,401,057	14,374	1,442	62,919	4,567	96.2%	5,241	4,262	4,865	243.81
LFU3 (FU6=DC5)	5'+VDJ	783,735,335	14,885	1,538	52,653	4,834	96.1%	6,114	5,259	3,539	353.04
LFU4 (FU7=DC6)	5'+VDJ	765,363,789	11,615	1,364	65,894	4,031	96.0%	5,700	4,567	3,525	70.20

Supplementary Table S3 The scRNA-seq / scTCR-seq protocol and data characteristics in the current study via Seurat package

Donor	ScRNA-seq Cell Counts	Filtered ScRNA Cells Counts by Quality Control	TRA Counts	TRB Counts	Paired TRA-TRB Cell Counts	Filtered TRA Counts	Filtered TRB Counts	Filtered Paired TRA-TRB Cell Counts	Filtered αβTCR-scRNA Cells Counts
HD1	11067	8093	4112	6137	3418	3530	5293	3125	2740
HD2	7099	6005	3318	4720	2698	2838	3996	2510	2323
HD3	8228	5733	3873	5054	3130	3209	4347	2888	2239
HD4	10016	7408	7417	9449	5158	6042	6881	4833	3852
HD5	11556	5636	7627	10557	5473	6595	7609	5203	2744
HD6	11607	6148	8607	11052	5948	7193	7647	5652	3476
DC1	12058	9484	7943	8702	5843	6809	7331	5597	5237
DC2	13965	12715	8034	9412	6023	6769	7759	5709	5446
DC3	9764	9468	6069	8763	4907	5148	7488	4565	4483
DC4	4271	3938	3492	4740	2936	3044	3249	2678	2034
DC5	6173	5653	5895	7996	5156	5128	6328	4543	3412
DC6	5919	4954	5938	7270	4946	5193	5815	4502	3428
EFU1 (FU1)	13911	13145	6569	7079	5128	5740	5983	4977	4824
EFU2 (FFU2)	12586	12052	3369	3575	2554	2972	2954	2485	2419
EFU3 (FU3)	21037	19791	4893	6276	3811	4236	5349	3634	3419
LFU1 (FU4)	10036	6014	13443	17106	9356	11290	13523	8802	4689
LFU2 (FU5=DC4)	5944	4092	6839	9268	4777	5866	6839	4556	2859
LFU3 (FU6=DC5)	7387	5517	8774	11475	5904	7286	8389	5578	3795
LFU4 (FU7=DC6)	6210	3885	7399	9866	5242	6380	7137	5006	2933

Supplementary Table S4 Performed scRNA-seq and paired TCR cell count in each group

		HD	Discharged	Follow-up
Raw Cells		59573	52150	77381
Filtered Cells by Quality Control		39023	46212	64496
Filtered αβTCR-scRNA Paired	Total cells	17374	24040	24938
	$CD3E^{+}CD4^{+}CD8A^{-}$ cluster cells	8718	13232	11021
	$CD3E^{+}CD4^{-}CD8A^{+}$ cluster cell	8354	10664	13227
Filtered Top20 αβTCR-scRNA Paired	Total cells	2902	2055	4854
	$CD3E^{+}CD4^{+}CD8A^{-}$ cluster cells	471	224	451
	$CD3E^{+}CD4^{-}CD8A^{+}$ cluster cell	2382	1809	4194
% of Filtered Top20 αβTCR Paired cells in Filtered Total αβTCR Paired Cells		16.7%	8.54%	19.46%

Supplementary Table S5 The unique-clone and multi-clone percentage in each group

Pt#	Unique-clone (1 cell)	Multi-clone (≥ 2 cells)
HD	67.13%	32.87%
Discharged	82.95%	17.05%
Follow-up	64.02%	35.98%

Unique-clone = (cell count of all unique TCR classification / total cell count) * 100%; Multi-clone = 100% - unique-clone.
